# Optimizing and evaluating the reconstruction of Metagenome-assembled microbial genomes

**DOI:** 10.1186/s12864-017-4294-1

**Published:** 2017-11-28

**Authors:** Bhavya Papudeshi, J. Matthew Haggerty, Michael Doane, Megan M. Morris, Kevin Walsh, Douglas T. Beattie, Dnyanada Pande, Parisa Zaeri, Genivaldo G. Z. Silva, Fabiano Thompson, Robert A. Edwards, Elizabeth A. Dinsdale

**Affiliations:** 1 Bioinformatics and Medical Informatics, San Diego State University, San Diego, California USA; 2 National Center for Genome Analysis Support, Indiana University, Bloomington, Indiana USA; 3 Department of Biology, San Diego State University, 5500 Campanile Drive, San Diego, 92115 California USA; 4 Computational Science Research Center, San Diego State University, San Diego, California USA; 5 Department of Biology, University of New South Wales, Sydney, New South Wales Australia; 6 Department of Mathematics and Statistics, San Diego State University, San Diego, California USA; 7 Institute of Biology, Federal University of Rio de Janeiro (UFRJ), Rio de Janeiro, Brazil; 8 Department of Computer Science, San Diego State University, 5500 Campanile Drive, San Diego, California USA

## Abstract

**Background:**

Microbiome/host interactions describe characteristics that affect the host's health. Shotgun metagenomics includes sequencing a random subset of the microbiome to analyze its taxonomic and metabolic potential. Reconstruction of DNA fragments into genomes from metagenomes (called metagenome-assembled genomes) assigns unknown fragments to taxa/function and facilitates discovery of novel organisms. Genome reconstruction incorporates sequence assembly and sorting of assembled sequences into bins, characteristic of a genome. However, the microbial community composition, including taxonomic and phylogenetic diversity may influence genome reconstruction. We determine the optimal reconstruction method for four microbiome projects that had variable sequencing platforms (IonTorrent and Illumina), diversity (high or low), and environment (coral reefs and kelp forests), using a set of parameters to select for optimal assembly and binning tools.

**Methods:**

We tested the effects of the assembly and binning processes on population genome reconstruction using 105 marine metagenomes from 4 projects. Reconstructed genomes were obtained from each project using 3 assemblers (IDBA, MetaVelvet, and SPAdes) and 2 binning tools (GroopM and MetaBat). We assessed the efficiency of assemblers using statistics that including contig continuity and contig chimerism and the effectiveness of binning tools using genome completeness and taxonomic identification.

**Results:**

We concluded that SPAdes, assembled more contigs (143,718 ± 124 contigs) of longer length (N50 = 1632 ± 108 bp), and incorporated the most sequences (sequences-assembled = 19.65%). The microbial richness and evenness were maintained across the assembly, suggesting low contig chimeras. SPAdes assembly was responsive to the biological and technological variations within the project, compared with other assemblers. Among binning tools, we conclude that MetaBat produced bins with less variation in GC content (average standard deviation: 1.49), low species richness (4.91 ± 0.66), and higher genome completeness (40.92 ± 1.75) across all projects. MetaBat extracted 115 bins from the 4 projects of which 66 bins were identified as reconstructed metagenome-assembled genomes with sequences belonging to a specific genus. We identified 13 novel genomes, some of which were 100% complete, but show low similarity to genomes within databases.

**Conclusions:**

In conclusion, we present a set of biologically relevant parameters for evaluation to select for optimal assembly and binning tools. For the tools we tested, SPAdes assembler and MetaBat binning tools reconstructed quality metagenome-assembled genomes for the four projects. We also conclude that metagenomes from microbial communities that have high coverage of phylogenetically distinct, and low taxonomic diversity results in highest quality metagenome-assembled genomes.

**Electronic supplementary material:**

The online version of this article (10.1186/s12864-017-4294-1) contains supplementary material, which is available to authorized users.

## Background

Microbiome studies describe the significance of microbial community that is associated with the host organism [1]. However, less than 1% of all microbial species can be cultured *in vivo* [2–4]; therefore, applications of culture-independent sequencing technology has revolutionized microbiome analysis [5–11]. Shotgun metagenomics provides a rapid assessment of microbial communities by sequencing a random subset of the genetic material from the environment [2, 6–10, 12]. Annotations of metagenomic DNA fragments is used to infer taxonomic and functional patterns within microbial communities across multiple environments, including oceans [7, 13], coral reefs [5, 9, 13–18], algae [19], and sharks [6]. However, linking the taxonomic origin of functional genes from metagenomes is a complex task, because the sequences belong to multiple genomes. In addition, many sequences may not match the database and therefore remain unidentified, for example in the viral community collected from a marine oxygen minimum zone only 2% of sequences were identified [20]. Improved sequencing technology and coverage have enabled reconstruction of fragments into metagenome-assembled genomes by process of assembly and binning. However, genome reconstruction is affected by sequencing technology and the biological characteristics of the microbial community. Sequencers are currently restricted by an inverse relationship between sequence length and the number of reads. Longer reads provide more accurate annotation, whereas, shorter reads produce greater coverage of the community. High coverage is preferred in diverse communities to identify rare species [21]. Similarly, if the divergence within the species in the metagenome is small, reconstruction of metagenome-assembled genomes will inherently become difficult due to the inseparability of the microbial genomes [2, 22]. It is unresolved how sequencing characteristics of read length and depth interact with the biological variation of the microbial community, during the reconstruction of genomes on real metagenomic datasets.

The first step in the reconstruction of genomes is assembly, where short metagenomic reads are joined based on sequence overlap to form longer sequences called contigs. Assemblers apply different algorithms which may influence reconstructed genome quality. Incorrect assembly draws ambiguous conclusions from the data and reduces the number of annotations [23]. Therefore, assembly evaluation is an important step that includes both contig continuity and contig chimerism. The program QUAST (Quality Assessment for Genome Assemblies) calculates contig continuity by describing both contig length and number of contigs [24]. Contig chimerism is due to random sequence overlap; therefore a contig contains sequences from divergent bacteria and can be removed by tools that assess read coverage like Bowtie [25]. While not often recognized, changes in species richness and evenness from raw sequences compared with assembled contigs can also be used to assess contig chimerism as assemblers should maintain richness (number of taxa identified) while increasing evenness (greatest with equal distribution of taxa) [26–28]. In addition, a substantial reduction in diversity may indicate chimera formation. Therefore, an optimal assembly will provide; a high number of long contigs, a high proportion of reads assembled, conserved species richness, and an increased species evenness.

**Fig. 1:**
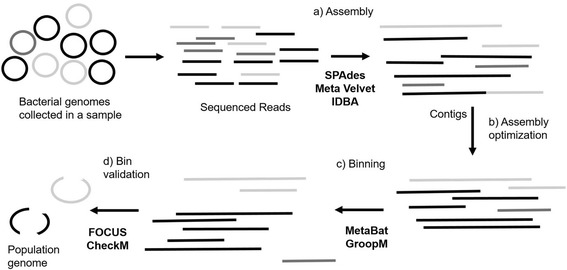
Overview of the workflow developed with the tools applied at each step (in bold). **a** Metagenomic reads are assembled using three assemblers: SPAdes, MetaVelvet, and IDBA. **b** Optimization of assembly tool using assembly statistics. **c** Assembled contigs from optimal assembly were binned using: MetaBat, and GroopM. **d** Optimal binning tool selected through bin validation. Colors (black, dark grey, and light grey) depict different microbial species, each line of a color representing the sequence belonging to a bacterial species

Binning reconstructs genomes of taxa from the individual contigs allowing for sequences with no homology to the databases to be annotated and taxonomic origin of functional genes to be identified [29–31]. Binning includes grouping phylogenetically related contigs into a bin, which represents a population genome containing the gene content of closely related species [32]. Binning tools group similar sequences based on sequence composition, which is an unsupervised approach that uses genomic signatures, such as GC content [33], tetranucleotide frequencies [34–36], and read coverage per contigs [2, 29, 30]. An ideal bin will represent one bacterial genome with minimal GC variation, species richness, and ~100% genome completeness. To increase the quality of binning, tools are advancing from applications using one genome signature, such as GroopM (group metagenomes) [30] and cross assembly [29], to applications using a combination of genome signatures, such as MetaBat (Metagenome Binning with Abundance and Tetra-nucleotide frequencies) [31]. The quality of the resulting bins is assessed by calculating the variation in GC content, species richness, and predicted genome completeness using tools, such as CheckM (check genome completeness) tool [37]. Bins containing sequences from mainly single taxa are metagenome-assembled genomes. Bins that contain sequences similar to multiple taxa, but include most of the bacterial marker genes may be novel population genomes. Identifying novel microbes is a crucial objective of reconstructing genomes from metagenomes. The phylogeny and genomic content of the novel genomes are investigated using tools such as CheckM [37], PhyloSift (phylogenetic analysis of genomes and metagenomes) [38], and RAST (Rapid Annotations using Subsystems Technology) [39]. Further, relatedness to species can also be identified using average nucleotide identity (ANI) that reciprocates the results from DNA-DNA hybridization experiments to show species relatedness [40]. In DNA-DNA hybridization a 70% cut-off delineates species relatedness and is reflected in the ANI calculations as the proportion of protein-coding regions that align between two genomes [41], if ANI is > 95%, it represents species relatedness [40]. As metagenomics analysis of microbial communities becomes more popular, many new genomic tools are being produced to analyze the DNA sequences (https://omictools.com *)*. There are benefits, and drawbacks of the analysis conducted by each tool and understanding how these analyses affect the results is essential to microbiologists. Previous evaluation of assemblers and binning tools have emphasized computational efficiency, including runtime, and memory usage. Many of these analyses were completed on synthetic microbial communities rather than actual metagenomic data [22], using parameters such as the number of miss-assemblies, genome recalls and precision that is a challenge to calculate on real datasets [22, 24, 31]. Another analysis has only used one assembler and binning tool [42], without comparing the effects of the assembler on the dataset. Other studies have spiked genomic reads into metagenomes to investigate the number of reads required to reconstruct a draft metagenomics-assembled genome [43]. In this paper, we investigate the effect of assembly and binning by comparing 105 metagenomes that were; 1) recovered from different marine environments, 2) varied in diversity, and 3) sequenced on different sequencing platforms. Biologically relevant parameters are used to analyze the data after the application of each tool. We hypothesize that the biological characteristics will affect assembly and binning. First, the assembly quality for the three assemblers: IDBA (Iterative De Bruijn graph Assembler), MetaVelvet (METAgenomic-Velvet assembler), and SPAdes (St. Petersburg genome assembler) was assessed using a set of assembly statistics, including contig continuity and contig chimerism. The most optimal assembler was applied to each project, followed by two composition based binning tools: GroopM and MetaBat to reconstruct genomes. These bins were assessed for genome completeness and taxonomic identification. Last, we explore the genomic content and phylogenetic relationships of a metagenome-assembled genome. Our pipeline is shown in Fig. 1.

## Methods

### Metagenomes collection

To test the effects of the assembly and binning processes on population genome reconstruction, we used 105 marine metagenomes from 4 projects. The projects were collected from coral atolls in Abrolhos Bank, Brazil (coral) and Southern California kelp forests (kelp) (see Additional file 1: Table S1). In two of the projects, the microbial community was experimentally manipulated before sequencing to reduce the diversity of the microbes, and these projects are labeled as coral low diversity (coral_IT_low) [9] and kelp low diversity (kelp_IL_low) [8]. The other two projects are natural microbial communities collected from the marine water associated the same environments and called coral high diversity (coral_IL_high) [14] and kelp high diversity (kelp_IT_high) [10]. Coral_IT_low and kelp_IT_high metagenomes were sequenced on Ion Torrent PGM (IT), 200 sequencing kit (ThermoFisher Scientific), whereas coral_IL_high, and kelp_IL_low was sequenced on an Illumina MiSeq v3 reagent cartridge (IL), 600 cycle kit (Illumina Inc.). Many metagenomes are publicly available on MG-RAST (MetaGenomics-Rapid Annotation using Subsystems Technology); thus the pipeline started with obtaining the metagenomes from this database [44](Table 1). The variation between the different projects was used to identify the repeatability of the workflow on datasets that vary with the environment from, level of biological diversity, and sequencing platform used.

**Table 1:** Background information on the projects used to evaluate the selection of assembly and binning tools

Project name	Source	Number of metagenomes	Total number of reads	Sequencing technology	Environment
coral_IL_high	Abrolhos, Brazil. 2014	16	20,711,400	Illumina MiSeq (IL)	Coral atolls (coral)
coral_IT_low	Abrolhos, Brazil. 2011	15	18,323,050	IonTorrent, PGM (IT)	Coral atolls (coral)
kelp_IL_low	San Diego, USA 2015	51	6,493,217	Illumina MiSeq (IL)	kelp forest (kelp)
kelp_IT_high	San Diego, USA 2012–2013	23	9,769,952	IonTorrent PGM (IT)	kelp forest (kelp)

The first step in a metagenomic pipeline is to remove poor quality sequences by running each metagenome through PRINSEQ (PReprocessing and INformation of SEQuence data) [45]. PRINSEQ was performed to remove sequencing tags, duplicates and N’s within the metagenome. Forward and reverse reads from Illumina MiSeq platform were first paired using PEAR (Paired-End Read merger) [46]. All the reads from a project were placed together in one file and cross-assembled (i.e., all metagenomes from the one project were assembled) using three De Bruijn graph assemblers: IDBA, MetaVelvet, and SPAdes. Default kmer sizes were applied for each tool; IDBA (k _min_: 25), MetaVelvet (kmer: 31) and SPAdes (kmers: 21, 33 and 55).

### Assembly evaluation

Each assembler (IDBA, MetaVelvet, and SPAdes) provides one output contig file for each project, therefore providing 12 contig files in total. We calculated the assembly statistics for the 12 contig files using QUAST [24], including N_50_ length, L_50_ (which includes the number of contigs longer than N_50_), the number of contigs assembled, the length of the largest contig, and the total length of the assembly. Contig continuity was assessed using contig length (length of 1000 contigs from 12 contig files), and the total number of contigs per assembly. Contig chimerism was first assessed by calculating the proportion of reads assembled (for 1000 contigs from 12 contig files) using Bowtie [25]. FOCUS (Find Organisms by Composition USage), a taxa identification tool that is alignment independent, was applied to the 12 contig files. The resulting information was used to calculate the Margalef richness and Pielou’s evenness of the 12 contig files using Primer statistics tool [47]. FOCUS was used explicitly for this step, as each contig is assigned to bacterial species based on kmer ratios [48]. Contig chimeras will have variable kmer ratios and will remain unidentified by Focus and be removed from further analysis. The second step for assessing contig chimerism included a comparison of Margalef richness and Pielou’s evenness of the 12 contig files against the metagenomic reads. The overall proportion of reads assembled into the entire assembly for the 12 contig files were also calculated using Bowtie.

The contigs from the optimal assemblers for each project were selected and uploaded to the Contig Clustering of Metagenomics (CCOM) tool [49] along with their read files in FASTA format to perform GroopM [30] and MetaBat [31] clustering. CCOM tool runs BWA (Burrows-Wheeler Aligner) aligner to map reads on contigs, the resulting output from the tools includes bam format. GroopM and MetaBat both use the contigs (.fasta) and reads (.bam) format as input to extract the resulting bins.

### Bin validation

CCOM tool extracted two sets of bins for GroopM and MetaBat binning tools for each project. Evaluation of binning tools was performed using bin characteristics including; variation in GC content, species richness and genome completeness. GC content was calculated using a self-written Biopython [50] script. Taxonomy composition for each bin was predicted using FOCUS [48]. Margalef’s species richness was calculated using Primer [47] for FOCUS taxonomy results. Genome completeness was assessed using CheckM [37]. A bin was identified as a specific population genome if the bins included sequences belonging to a single genus. Species or strain level resolution could be used depending on the amount of coverage and diversity of the microbes. Potentially novel bins were identified as those bins that contained > 50% genome completeness but were not annotated by FOCUS. These potentially novel bins were further analyzed using CheckM [37], PhyloSift’s [38], and RAST [39], all of which predict the neighboring genomes using marker genes. Proteome content of a novel population genome was investigated using PATRIC (Pathosystems Resource Integration Center) [51], followed by calculating the average nucleotide identity of the protein-encoding genes by applying the blast (ANIb) analysis and tetranucleotide correlation search (TCS) in JSpeciesWS tool [41].

### Statistical analysis

The first statistical analysis was a one-way ANOVA (ANalysis Of VAriance) conducted on the unassembled metagenomes from each project to identify differences in microbial diversity. Assembly evaluation variables included the number of contigs, richness, and evenness, and binning tools evaluation variables included, GC content, species richness, and genome completeness. These variables were tested for normality using the Shapiro-Wilks test, and non-normal data was log transformed when appropriate. Data containing many instances (> 5000), for example, contig length and percent of reads assembled, were tested for normality using the Kolmogorov-Smirnov test and non-normal data was log transformed when appropriate. To test for differences in assemblers, a one-way ANOVA was conducted on the following variables; the number of contigs, richness, and evenness. A one-way ANOVA was used because there was only one data point for each variable per project because the metagenomes were cross assembled. To investigate whether the assemblers performed differently depending on the projects a 2-way ANOVA model was conducted on the factors; project, assemblers and projects by assemblers as the interaction term for the variables contig length and reads assembled. For the 2-way ANOVA, the data was subsampled to select for the 1000 longest contigs in each project, because running statistics on all 300,000 contigs is not feasible. Tukey HSD post hoc comparisons were performed to identify the project that contributed to the differences. Similar statistics were conducted on the binning evaluation variables for the two binning tools, MetaBat and GroopM. Therefore, to investigate whether the binning tools performed differently depending on the projects a 2-way ANOVA model was conducted on the factors; project, binning tools, and projects by binning tools as the interaction term for the variables; GC variation, richness and genome completeness. Overall, the statistical analysis was implemented using R scripts and visualized using Sigma Plot (Systat Software, San Jose, CA).

## Results

### Variation between projects

The metagenomes from four projects were downloaded from MG-RAST (Table 1). Samples were from two environments; coral atolls and kelp forest, sequenced on two sequencing platforms; Illumina and IonTorrent (Table 1). In each environment, a subset of samples was experimentally manipulated before sequencing to reduce the diversity of the microbes. Diversity measures were significantly different between the four projects (*P* < 0.05) (see see Additional file 2: Figure S1). Tukey HSD post hoc conducted on the four diversity parameters showed that the coral_IT_low project was significantly lower in diversity from the remaining projects (*P* < 0.05) (see Additional file 3: Table S2). However, the manipulation of the kelp_IL_low project did not result in a significant decrease in taxonomic diversity.

### Assembly evaluation

The 12 contig files (4 projects, 3 assemblers) were analyzed using QUAST, which identified that SPAdes and IDBA provided high contig continuity compared to MetaVelvet that assembled fewer contigs, with short contig lengths (see Additional file 4: Table S3).

**Fig. 2:**
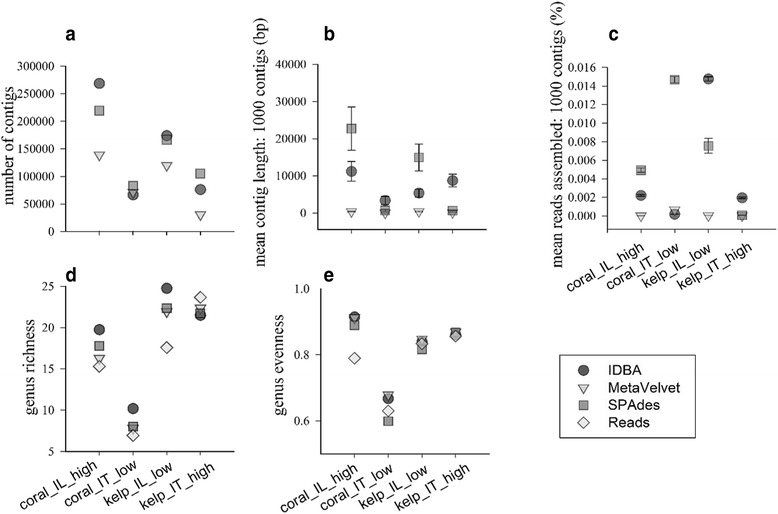
Assembly evaluation of IDBA, MetaVelvet, and SPAdes assemblers for cross assembled contigs for the four projects: coral_IL_high, coral_IT_low, kelp_IL_low and kelp_IT_high based on parameters; (**a**) number of contigs, (**b**) mean contig length for1000 contigs (bp), (**c**) mean reads assembled for 1000 contigs (%), (**d**) richness, and (**e**) evenness. Here we show the performance of each assembler in terms of all the five parameters for each project, the lines in graph b and c represent the standard errors

Contig continuity was further assessed using contig length (length of 1000 contigs from 12 contig files), the total number of contigs per assembly, and by calculating of proportion of reads assembled (1000 contigs from 12 contig files). Each project assembled a significantly different number of contigs (*F*_3, 8_ = 6.56, *P* = 0.01), greater number of contigs were assembled for Illumina (coral_IL_high = 209,144 ± 26,756, kelp_IL_low = 153,607 ± 11,954) compared to IonTorrent (coral_IT_low = 73,772 ± 3450, kelp_IT_high = 70,759 ± 15,380) (Fig. 2a). The length of 1000 contigs from the 12 files showed a significant difference between the three assemblers (*F*_2, 11,994_ = 133,077, *P* < 0.001), four projects (*F*_3, 11,994_ = 35,061, *P* < 0.001) and an interaction between the projects and assemblers (*F*_6, 11,994_ = 7551, *P* < 0.001). SPAdes provided longer contig for Illumina (coral_IL_high: 22,728 ± 5797 bp, kelp_IL_low: 14,957 ± 3660 bp) compared to IonTorrent projects (coral_IT_low: 697 ± 299 bp, kelp_IT_high: 638 ± 51 bp) (Fig. 2b). IDBA assembler performed uniformly for the different projects varying from a mean length of 3359 bp to 11,203 bp. A Tukey HSD post hoc test showed that all the project and assembler combinations were significant (see Additional file 5: Table S4).

Contig chimerism was assessed using Bowtie analysis which identifies the number of reads in the assembly by mapping the reads to contigs. Significant differences were observed for reads assembled (1000 contigs) between assemblers (*F*_2, 11,988_ = 29,139, *P* < 0.001), projects (*F*_3, 11,988_ = 4677, *P* < 0.001), and the interaction term between assemblers and projects (*F*_6, 11,988_ = 8046, *P* < 0.001) (see Additional file 6: Table S5). The differences were caused by the high diversity samples having a lower proportion of reads assembled (coral_IL_high, kelp_IT_high) compared with the low diversity samples (coral_IT_low, kelp_IL_low) having a higher proportion of reads assembled (Fig. 2c). IDBA and SPAdes followed this pattern except for IDBA coral_IT_low samples which assembled a lower number of reads (Fig. 2c). SPAdes were found to be selective for coral atoll projects (coral_IL_high, coral_IT_low) providing contigs with a higher read coverage compared to kelp forest samples (kelp_IL_low, kelp_IT_high) (Fig. 2c).

The richness and evenness of the assembled sequences were compared against their respective unassembled reads and showed no significant difference in diversity after assembly (richness; *P* = 0.92, evenness; *P* = 0.91), suggesting that microbial richness was maintained with minimal chimera formation. Similarly, microbial evenness did not show a significant difference between assemblers (Fig. 2) (see Additional file 7: Table S6).

Overall the assessment showed that the SPAdes assembly generated contigs of longer length (N_50_: 1632 bp) with a higher proportion of reads assembled into contigs (reads assembled (all contigs): 19.65 ± 1.41%) compared with IDBA (N_50_: 1024 ± 7.15 bp, reads assembled (all contigs): 16.83 ± 1.56%). However, SPAdes assembler performed selectively for the different projects (Fig. 2), suggesting that the underlying biology and sequencer affect assembly. The assembly provided by IDBA was similar across all projects, suggesting it is not responsive to the underlying biology of the microbial communities. MetaVelvet performed poorly in all aspects. In addition, SPAdes assembly showed no significant bias in richness and evenness compared to the reads, suggesting the lower proportion of contig chimerism. Therefore, based on our data of contig continuity and contig chimerism, we selected SPAdes as the optimal assembler.

### Binning tools evaluation

SPAdes assembled contigs for the four projects were binned using two different binning tools, GroopM and MetaBat. The GroopM binning tool applies only one genome signature: contig coverage, i.e. it groups contigs that have a similar proportion of reads that were combined from each metagenome, and this process extracted a high number of bins (coral_IL_high: 71, coral_IT_low: 31, kelp_IL_low: 117, and kelp_IT_high: 37 bins). MetaBat applies a combination of two genome signatures, contig coverage and tetranucleotide frequency, and the more stringent parameters extracted less bins (coral_IL_high: 57, coral_IT_low: 17, kelp_IL_low: 17, and kelp_IT_high: 24 bin).

**Fig. 3:**
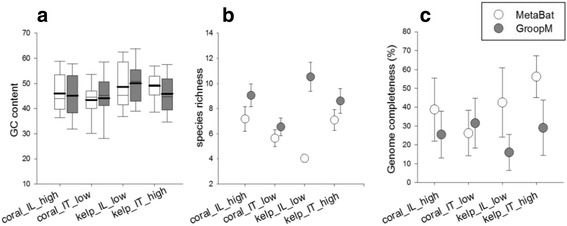
Evaluation of binning tools MetaBat (white) over GroopM (grey) using three parameters, (**a**) variation in GC content, shown as a box and whisker plot where the mean value is represented by a bold line in the box, the second line represents the median value for the data, (**b**) species richness, and (**c**) genome completeness (%). Error bars are one standard error

The population genome bins obtained from GroopM and MetaBat were evaluated for the following parameters; variation in GC content, genus richness and genome completeness (Fig. 3a). Two-way ANOVA was performed on variation in GC content, genus richness and genome completeness and identified differences between binning tools (GC variation: *F*_1, 368_ = 4.43, *P* < 0.03, genus richness: *F*_1, 362_ = 37.56, *P* < 0.001, genome completeness: *F*_1, 367_ = 24.78, *P* < 0.001). Significant interaction between the projects and binning tools was detected for parameters: GC variation (*F*_3, 368_ = 19.18, *P* < 0.001), richness (*F*_3, 362_ = 4.96, *P* < 0.001) and genome completeness (*F*_3, 367_ = 3.88, *P* < 0.001). MetaBat produced bins from the low diversity coral reef, and kelp forest projects are each dominated by one or a few species, showing that low diversity samples separate into better population genomes. The bins extracted from GroopM for the kelp low diversity were poorly separated with multiple taxa identified in each bin (Fig. 3b). For genome completeness, MetaBat bins contained greater completeness compared with GroopM for all the projects, except for coral_IT_low (Fig. 3c). Overall, MetaBat produced bins with less variation in GC content, low species richness (4.91 ± 0.66), and higher genome completeness (40.92 ± 1.75) compared to GroopM (species richness: 7.41 ± 0.66, genome completeness: 25.17 ± 1.80) (see Additional file 8: Table S7) irrespective of the project (Fig. 3).

### Bin validation and metagenome-assembled genome identification

**Fig. 4:**
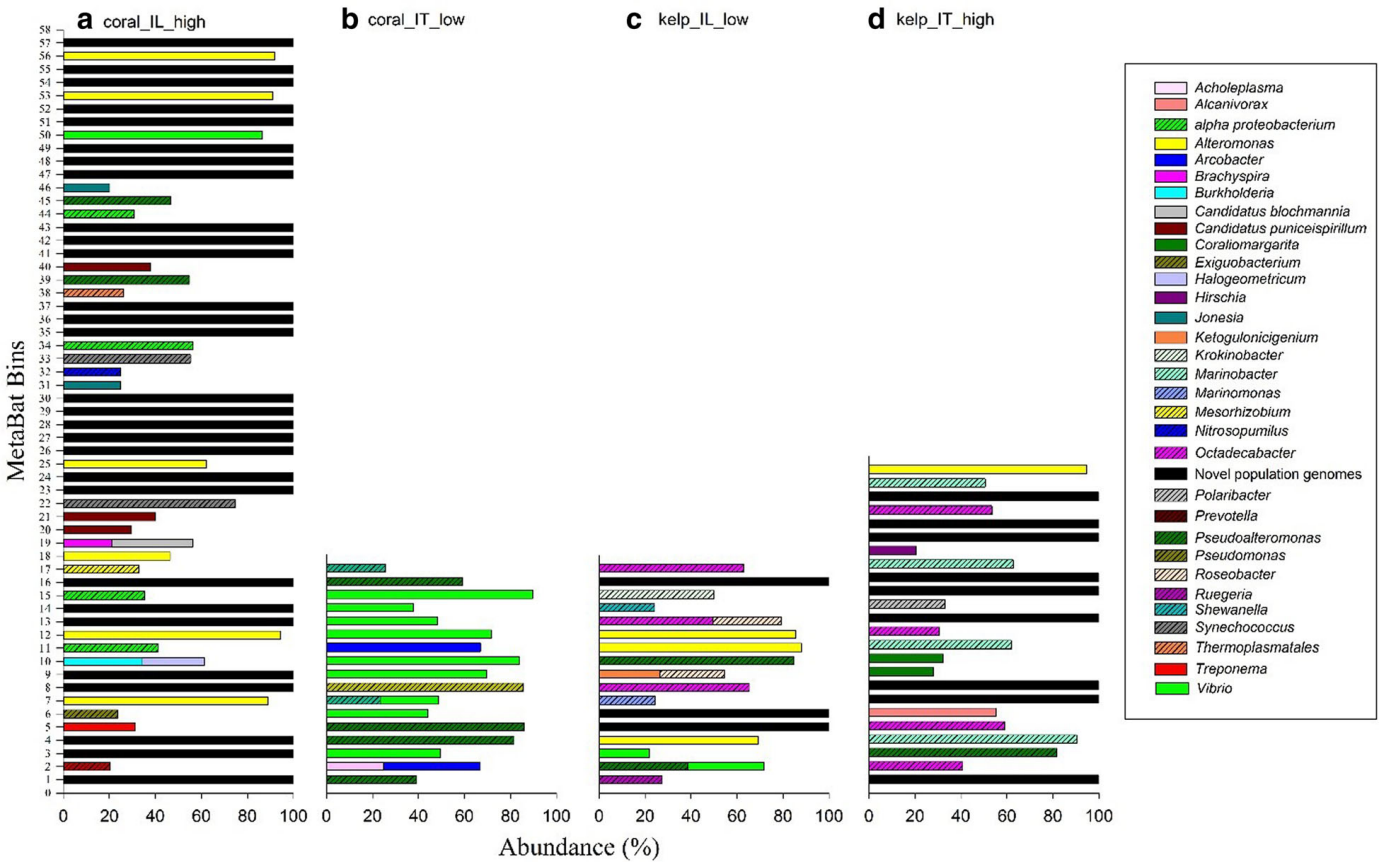
Taxonomic identification of the MetaBat bins using FOCUS for the four projects; (**a**) coral_IL_high, (**b**) coral_IT_low, (**c**) kelp_IL_low and (**d**) kelp_IT_high. Population genomes belonging to the 32 genera have been identified with abundance (> 20%) and their relative abundance in a bin is plotted. We also include a category “potentially novel population genomes” in black to represent bins that were identified to different taxa with low abundance. We predict that the bins with high species richness and have greater than 50% genome completeness are potentially novel population genomes

An ideal reconstruction of a microbial genome would be where each bin represents one metagenome-assembled genome that includes a high abundance of contigs of closely related species. Therefore, the taxonomic composition of the MetaBat bins was identified using FOCUS, because these are reconstructed genomes from metagenomics data, some of the contigs that are placed into a bin may not have a taxonomic annotation, and these contigs will represent novel genomic material from the environment. In addition, some of the contigs that are placed in similar bins will have mixed taxonomic assignments, suggesting that these contigs have come from phylogenetically similar organisms to those in the database, which cannot be separated by this process. In some bins, most contigs will have a similar taxonomic identification, with a few contigs that are from distinct taxa, and these could be DNA that has been horizontally transferred or contamination by contigs that cannot be sorted by the binning process. Identifying novel organisms, sister species, and horizontal gene transferred DNA is an important part of the reconstruction process and will increase the description of microbial diversity. Each project produced a different proportion of metagenome-assembled genomes that were similar to a single genus; coral_IL_high showed 46.42%, coral_IT_low showed 88.23%, kelp_IL_low showed 64.70% and kelp_IT_high showed 62.5% (Fig. 4). Genus level classification was applied to identify closely related species. Kelp_IL_low bin 9, and bin 13 contained multiple genera, *Ketogulonicigenium*, *Ruegeria*, and *Roseobacter*, suggesting these bins contain sequences belonging to family Rhodobacteraceae and thus could represent closely related novel species. Several bins contained a high abundance of sequences belonging to one microbial genus (*Alteromonas* or *Vibrio* metagenome-assembled genomes), however, they also included sequences belonging to other distantly relates taxa. A proportion of bins from each project had high completeness, but the genus identification was not apparent through FOCUS, suggesting they could be potential novel genomes (shown in black in Fig. 4). The proportion of potentially novel genomes varied depending on projects, for example, coral_IT_low showed no potentially novel genomes, and coral_IL_high had 51.78% of potentially novel metagenome- assembled genomes.

### Investigating novel metagenome-assembled genome

Overall, 13 bins (coral_IL_high: 7 bins, kelp_IL_low: 1 bin, and kelp_IT_high: 5 bins) had ≥ 50% completeness with ambiguous genus identifications (Table 2). These bins contain sequences with similar tetranucleotide frequencies, similar contig coverage profiles, and high genome completeness (presence of bacterial marker genes). The 13 potentially novel metagenome-assembled genomes were analyzed using marker genes and alignment to identify their closest phylogenetic neighbors using CheckM, PhyloSift, RAST, and ANI (Table 2). From the 13 bins, 8 bins were identified by two or more tools as the same microbial species, coral_IL_high bin 13 contains sequences belonging to class Alphaproteobacteria, coral_IL_high bin 14 is phylogenetically similar to *Alteromonas* genus, coral_IL_high bin 41 to marine gamma proteobacterium, coral_IL_high_54 to SAR86 cluster, kelp_IL_low bin 5 to *Oceanibulbus indolifex*, kelp_IL_low bin 8 to *Limnobacter sps*, kelp_IL_low bin 7 to belong to order Flavobacteriales and kelp_IL_low bin 20 to belong to family Rhodobacteraceae (Table 2).

**Table 2:** List of 13 novel bins identified from the four projects, the closest neighbor with similarity index using CheckM, PhyloSift, RAST, and JSpeciesWS

Project	Number of contigs	Completeness	GC (%)	Genome size (Mbp)	Gene count	CheckM	PhyloSift	RAST	JSpeciesWS (best hit that has >90%)
coral_IL_high_13	769	100	53.7	3.96	4342	*Parvibaculum lavamentivorans*	Alphaproteobacteria strain IMCC14465	*Parvibaculum lavamentivorans*	*Pelagibacter ubique*
coral_IL_high_14	1215	99.14	44.3	8.85	7897	–	Alphaproteobacteria strain HIMB5	*Alteromonas macleodii*	*Alteromonas mediterranea*
									*Alteromonas naphthalenivorans*
coral_IL_high_26	1554	87.93	34.6	6.72	7756	Verrucomicrobia	SAR86 cluster bacterium SAR86A	Ruegeria sp. R11, Roseobacter denitrificans OCh 114	*Alteromonas mediterranea*
coral_IL_high_28	270	52.27	39.4	1.03	1175	*Alteromonas taeanensis*	Flavobacteria strain MS024 2A	Polaribacter sp. MED152	SAR116 cluster alpha proteobacterium HIMB100
coral_IL_high_41	825	79.67	56.7	2.99	3384	–	Gammproteobacteria strain HIMB55	marine gammaproteobacteria strain HTCC2080	–
coral_IL_high_49	3536	50.62	51.1	12.97	12,794	Bacteria	–	Gammaproteobacteria strain IMCC3088	–
coral_IL_high_54	297	93.1	37.8	2.51	2776	unresolved	SAR86 cluster strain SAR86E	SAR86 cluster bacterium SAR86E	–
kelp_IL_low_5	1046	90.7	62.8	5.16	5903	*Oceanibulbus indolifex*	*Oceanibulbus indolifex*	*Oceanibulbus indolifex* HEL-45	–
kelp_IT_high_1	848	82.45	51.6	5.04	7180	*Rubritalea marina*	Verrucomicrobia strain SCGC AAA168 F10	*Akkermansia muciniphila*, *Verrucomicrobium spinosum* DSM 4136	*Marinobacter salarius*
									*Marinobacter algicola*
kelp_IT_high_7	487	77.27	43.3	1.97	3260	*Owenweeksia hongkongensis*	Flavobacteria strain MS024 2A	*Kordia algicida*OT-1	–
kelp_IT_high_8	1224	54.55	52.1	2.98	5637	*Limnobacter*	*Limnobacter sp.*MED105	*Limnobacter sp.*MED105	*Marinobacter sps*
kelp_IT_high_16	1011	57.94	39.5	2.06	3981	Flavobacteriaceae	SAR86 cluster strain SAR86C	Tenacibaculum sp. MED152	–
kelp_IT_high_20	836	84.8	52	4.19	6227	Rhodobacteraceae	Rhodobacteraceae strain HTCC2150	*Roseovarius nubinhibens*	–

### Distinguishing novel metagenome-assembled genomes

**Fig. 5:**
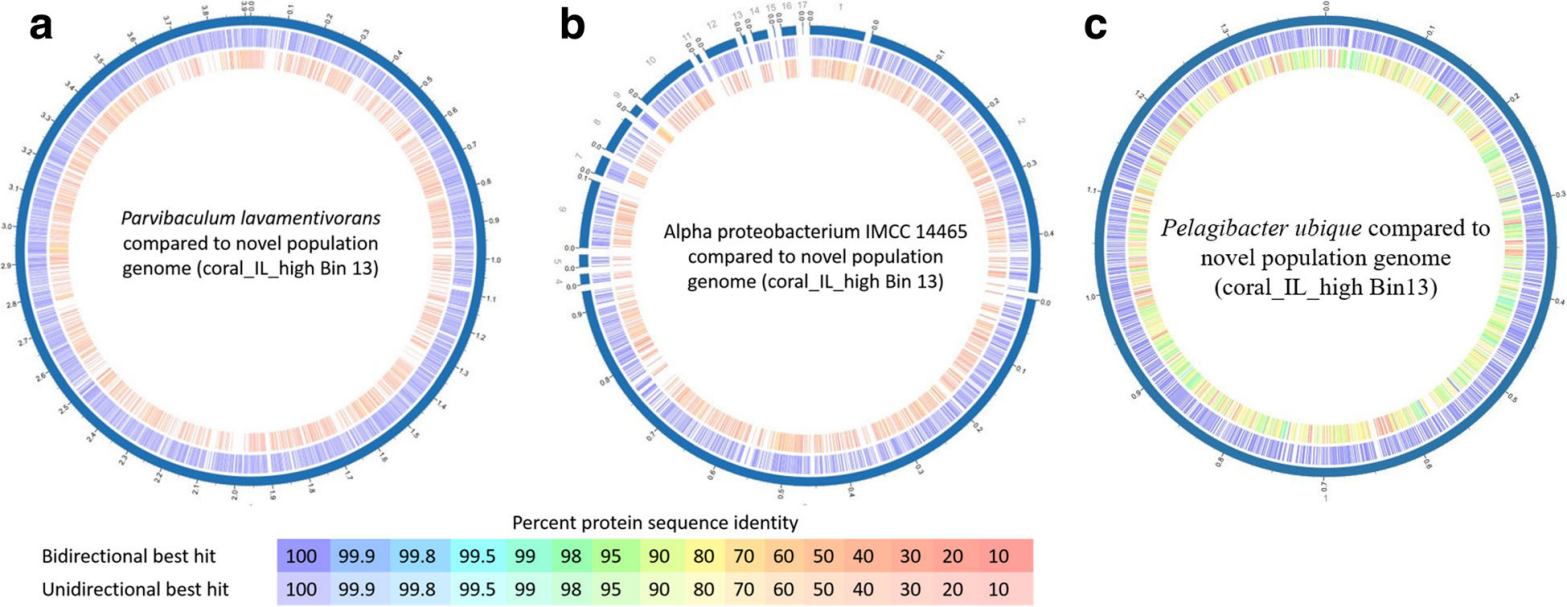
Proteome comparison of the reconstructed population genome (coral_IL_high Bin 13) compared against the genomes closest neighbors *Parvibaculum lavamentivorans* (**a**), Alpha proteobacterium IMCC14465 (**b**) and *Pelagibacteria ubique* (**c**). The outer ring represents the contig of the reference species. The middle ring represents the reference bacterial species, and the inner most ring represent the potentially novel population genome with the color scale representing the protein similarity

A single metagenome-assembled genome; coral_IL_high bin 13 was identified to have 100% genome completeness, containing all 104 conserved bacterial marker genes. The metagenome-assembled genome was phylogenetically affiliated with *Parvibaculum lavamentivorans*, by CheckM and RAST, and Alpha proteobacterium IMCC 14465 by PhyloSift. Using GC content, genome size, the number of protein-encoding genes, and the number of RNA genes the reconstructed genome (coral_IL_high bin 13) was more similar to *Parvibaculum lavamentivorans* compared with Alphaproteobacteria IMCC14465 (see Additional file 9: Table S8). However, the proteome of the reconstructed genome compared to *Parvibaculum lavamentivorans* and Alphaproteobacteria IMCC 14465 showed 44.12% similarity to both the reference organisms (Fig. 5a and b). Average nucleotide identity (ANI) of the novel population genome was calculated to show 63.50% similarity with Alphaproteobacteria IMCC14465, and 62.52% similarity with *Parvibaculum lavamentivorans*. The tetranucleotide frequencies of the novel metagenome-assembled genome were further compared against a database to be 82.22% similar to *Pelagibacter ubique.* Proteome comparison against *Pelagibacter ubique* showed to have 90.35% (Fig. 5c) compared to the 44.12% shown earlier (Fig. 5b). Coral_IL_high bin 13 contains twice as high GC content, genome size, the number of protein-encoding genes, and RNA sequences compared with *Pelagibacter ubique* (see Additional file 9: Table S8), we suggest it is a novel genome within the Alphaproteobacteria. The identification of the novel genomes provides support that the metagenome-assembled genomes contain environmentally relevant genomic material that is not in the cultured relatives from the databases.

## Discussion

We present a set of evaluation parameters to optimize the workflow to reconstruct metagenome-assembled genomes from environmental microbial communities using assembly evaluation parameters; the number of contigs, contig length, the proportion of reads assembled, genus richness, evenness and binning evaluation parameters; GC content, species richness, and genome completeness. Selection of the four projects, containing 105 metagenomes, in the study accounts for variation in biological and procedural biases that are common in every microbiome study. By including these variables in the optimization, rather than using mock communities or few metagenomes [22, 26, 31, 43, 52], we tested the tools under realistic conditions and identified biases. For our datasets, SPAdes assembler and MetaBat binning tools provided optimal results, and our evaluation techniques could be used to explore and evaluate new assemblers and binning tools.

### Assembly evaluation parameters

The metagenomic variations within the projects influenced the performance of the assemblers. To select an optimal assembler, contig length, the number of contigs, and proportion of reads assembled showed that MetaVelvet performed poorly and was not considered further. The underlying algorithm for both IDBA and SPAdes assemblers apply *De-Bruijn* graphs. The difference included, IDBA iteratively improving the kmer size based on input [28, 52], and SPAdes sequentially assembling the metagenomes with kmer fragments between 21 to 127 [27].We observed that SPAdes assembled contigs were longer for Illumina samples compared to IonTorrent samples. We predict as the SPAdes assembler further fragments the reads to different kmer sizes to form contigs, the overlapping region between forwards and reverse reads from Illumina facilitates the forming of longer contigs [27]. More reads were incorporated to contigs for coral environment samples when using SPAdes and for kelp forest samples when using IDBA, which could be due to the bias associated with the algorithms in handling the variability within the microbial communities. We included two additional parameters, species richness and evenness to account for shortcuts applied in the assembly algorithms that include a data reduction step to discards the low abundant sequences, and formation of contig chimeras [53]. A decrease in species richness compared to the unassembled metagenomes would suggest contig chimeras. However, all assemblies showed a slight increase in species richness, and conserved evenness suggesting minimal contig chimeras were constructed by IDBA or SPAdes. IDBA assembler performance was more uniform suggesting that the assembler is treating all datasets the same and does not take advantage of underlying structure in the metagenomes, such as longer reads. The IDBA documentation is minimal [52], and this may affect the user’s ability to use the assemblers to full potential. In conclusion, the applied parameters showed SPAdes assembly provides the best contig continuity and minimal contig chimerism across four different microbial environments and displayed flexibility with each of the biological and platform biases. While conducted on far less data, other studies have also found SPAdes to provide longer contigs with more reads used in the assembly [26, 52].

### Binning tool selection

MetaBat was selected as the optimal binning tool because the bins had minimal GC variation, species richness, and high genome completeness that may represent a single genome. The number of bins extracted by MetaBat was low compared to GroopM extracted bins. MetaBat bins were further validated using taxonomic identification to show the workflow reconstructed 66 metagenome-assembled genomes. These metagenome-assembled genomes include sequences of closely related species; therefore, they were identified to the genus level. Each metagenome-assembled genome contained sequences belonging to distant bacterial species, suggesting possible horizontal gene transfers or novel sequences with no genome relative in the database. Metagenome-assembled genomes of *Arcobacter* extracted from coral reefs were studied to identify unique genes that were previously not associated with the genomes cultured from other environments [9]. Identification of potentially novel genomes extracted from metagenomes relies on the presence of marker genes [32, 54]. A novel population bin (coral_IL_high bin 13) that has all the bacterial genome markers used in CheckM, and was phylogenetically affiliated to the bacterial species *Parvibaculum lavamentivorans*, with 44% proteome similarity using Focus. Further analysis with ANI and JSpeciesWS (TCS), suggested 82.22% similarity to *Pelagicater ubique*. ANI > 95% represents over 70% DNA-DNA hybridization which shows species relatedness, suggesting that Bin 13 falls below the species levels classification. The conflicting results of two kmer based tools, suggests that the genomes are novel and therefore do not closely match organisms in the databases. In addition, several databases need to be used in the description of metagenome-assembled genomes to overcome any database bias. The resulting metagenome-assembled genomes enable linking taxa to function to understand the role of the population in the microbial community, and we are currently investigating the role of these genomes in the coral reef environment [14]. Our pipeline meets the minimum standards for metagenome-assembled genomes [55]. In the process, novel genomes, genes, and sequences were identified, which can now be deposited in a database to improve future annotation [29, 32, 56].

## Conclusions

We present a set of assembly and binning evaluation parameters to select for an optimized workflow to reconstruct metagenome-assembled genomes (see Additional file 10). The set of parameters provides biologically relevant information regarding richness, evenness, and GC content to help infer the optimal tools for the dataset. Using these parameters, we present an optimized workflow for four metagenome projects, to be SPAdes assembly and MetaBat binning tool regardless of the metagenomic variations. However, the metagenomic variations within each project did result in the differential quality of the metagenome-assembled genomes. Communities that have high coverage of phylogenetically distinct organisms and low taxonomic diversity resulted in better quality genome reconstruction.

## Additional files

Additional file 1: Table S1. Metagenomes used in this study. List of metagenomes used in the analysis and the sequencing statistics. (DOCX 20 kb)

Additional file 2: Figure S1. Microbial diversity in the 4 microbiome projects. Representation of microbial diversity using, (a) genus richness, (b) genus evenness, (c) Shannon diversity, and (d) Simpson diversity of the four projects, which are represented on the x axis. The box represents 50% of the data ranges around the median. The outliers for each case are represented as black dots. (DOCX 167 kb)

Additional file 3: Table S2. Post hoc Tukey HSD test results for diversity analysis. Post hoc Tukey HSD test results for Shannon, Simpson, Richness and Evenness for the four projects. (DOCX 14 kb)

Additional file 4: Table S3. Assembly statistics. QUAST results for the 12 Contigs files assembled using the three assemblers; IDBA, MetaVelvet, SPAdes. (DOCX 16 kb)

Additional file 5: Table S4. Post hoc Tukey HSD test results for contig length. Post hoc Tukey test results comparing contig length of 1000 contigs across assemblers and projects. (DOCX 18 kb)

Additional file 6: Table S5. Post hoc Tukey HSD test results for mean reads assembled. Post hoc Tukey test results for the mean reads assembled (%) of 1000 contigs across assemblers and projects. (DOCX 18 kb)

Additional file 7: Table S6. Assembly evaluation parameters. List of all the assembly evaluation parameters. (DOCX 16 kb)

Additional file 8: Table S7. Binning tool evaluation parameters. List of the parameters for the GroopM and MetaBat extracted bins. (DOCX 51 kb)

Additional file 9: Table S8. Comparison on metagenome-assembled genomes. Comparison of the genome parameters of novel metagenome-assembled genome (coral_IL_high Bin 13) against the three closest genomes from the database. (DOCX 13 kb)

Additional file 10: Optimized workflow. Guide to optimized workflow to reconstruct metagenome-assembled genomes. Description of the programs used in this study at each step and the evaluation parameters calculation is provided as step by step workflow. (DOCX 148 kb)

## Abbreviations

ANI: Average Nucleotide Identity
ANOVA: ANalysis Of VAriance
BWA: Burrows-Wheeler Aligner
CCOM: Contig Clustering of Metagenomics
CheckM: Check genome completeness
coral_IL_high: coral high diversity
coral_IT_low: coral low diversity
FOCUS: Find Organisms by Composition USage
GroopM: Group Metagenomes
IDBA: Iterative De Bruijn graph Assembler
IL: Illumina MiSeq
IT: Ion Torrent PGM
kelp_IL_low: kelp low diversity
kelp_IT_high: kelp high diversity
MetaBat: Metagenome Binning with Abundance and Tetra-nucleotide frequencies
MetaVelvet: (METAgenomic-Velvet assembler)
MG-RAST: MetaGenomics-Rapid Annotation using Subsystems Technology
PATRIC: Pathosystems Resource Integration Center
PEAR: Paired-End Read merger
PhyloSift: Phylogenetic analysis of genomes and metagenomes
PRINSEQ: PReprocessing and INformation of SEQuence data
QUAST: Quality Assessment for Genome Assemblies
RAST: Rapid Annotations using Subsystems Technology
SPAdes: (St. Petersburg genome assembler)
TCS: Tetranucleotide Correlation Search
