# Supplementary material for: Optimizing and evaluating the reconstruction of Metagenome-assembled microbial genomes
Source: BMC Genomics. 2017 Nov 28;18:915. doi: 10.1186/s12864-017-4294-1 (PMC5706307; doi:10.1186/s12864-017-4294-1)
Supplement: Supplementary file 1 — Metagenomes used in this study. List of metagenomes used in the analysis and the sequencing statistics. (DOCX 20 kb) [file 12864_2017_4294_MOESM1_ESM.docx]

Supplementary Table 1. List of metagenomes used in the analysis and the sequencing statistics.

|  | | | | | |
| --- | --- | --- | --- | --- | --- |
| Project name | **Metagenomes** | **MGRAST ID** | **Number of sequences** | **Average read length** | **Sequencing Platform** |
| coral_IL_high | algae1.2 | 4618073.3 | 416,747 | 294 | Illumina MiSeq (IL) |
| coral_IL_high | algae3a | 4618074.3 | 163,809 | 310 | Illumina MiSeq (IL) |
| coral_IL_high | algae4a.1 | 4618075.3 | 935,817 | 309 | Illumina MiSeq (IL) |
| coral_IL_high | coral1.1 | 4618077.3 | 1,548,300 | 236 | Illumina MiSeq (IL) |
| coral_IL_high | coral2b.1 | 4618078.3 | 1,442,579 | 250 | Illumina MiSeq (IL) |
| coral_IL_high | coral3.1 | 4618738.3 | 5,172,040 | 269 | Illumina MiSeq (IL) |
| coral_IL_high | coral2.2 | 4618079.3 | 218,453 | 295 | Illumina MiSeq (IL) |
| coral_IL_high | algae_cyano | 4618076.3 | 1,590,789 | 265 | Illumina MiSeq (IL) |
| coral_IL_high | cyano2a | 4618080.3 | 1,577,993 | 265 | Illumina MiSeq (IL) |
| coral_IL_high | cyano3a | 4618081.3 | 1,272,509 | 315 | Illumina MiSeq (IL) |
| coral_IL_high | wc1.1 | 4618082.3 | 2,210,322 | 243 | Illumina MiSeq (IL) |
| coral_IL_high | wc1.2 | 4618083.3 | 271,479 | 307 | Illumina MiSeq (IL) |
| coral_IL_high | wc2.2 | 4618084.3 | 245,008 | 302 | Illumina MiSeq (IL) |
| coral_IL_high | WC2b | 4618445.3 | 2,488,563 | 265 | Illumina MiSeq (IL) |
| coral_IL_high | zoo1.2 | 4618085.3 | 472,699 | 276 | Illumina MiSeq (IL) |
| coral_IL_high | zoo2.1 | 4618086.3 | 684,293 | 238 | Illumina MiSeq (IL) |
| coral_IT_low | Algae_11 | 4661025.3 | 514323 | 223 | Ion Torrent PGM (IT) |
| coral_IT_low | Algae_12 | 4661568.3 | 1000211 | 232 | Ion Torrent PGM (IT) |
| coral_IT_low | Algae_13 | 4661570.3 | 1286243 | 235 | Ion Torrent PGM (IT) |
| coral_IT_low | Algae_14 | 4661564.3 | 1461897 | 194 | Ion Torrent PGM (IT) |
| coral_IT_low | CCA_11 | 4661141.3 | 1543336 | 226 | Ion Torrent PGM (IT) |
| coral_IT_low | CCA_12 | 4661140.3 | 1218588 | 202 | Ion Torrent PGM (IT) |
| coral_IT_low | CCA_13 | 4661139.3 | 222315 | 217 | Ion Torrent PGM (IT) |
| coral_IT_low | Control_11 | 4662101.3 | 1810513 | 245 | Ion Torrent PGM (IT) |
| coral_IT_low | Control_12 | 4662094.3 | 2101082 | 230 | Ion Torrent PGM (IT) |

(table continues)

**Supplementary Table 1. (continued)**

| coral_IT_low | Control_13 | 4662093.3 | 765762 | 206 | Ion Torrent PGM (IT) |
| --- | --- | --- | --- | --- | --- |
| coral_IT_low | Control_14 | 4662092.3 | 746457 | 223 | Ion Torrent PGM (IT) |
| coral_IT_low | Coral_11 | 4696550.3 | 1623787 | 233 | Ion Torrent PGM (IT) |
| coral_IT_low | Coral_12 | 4696548.3 | 1459735 | 237 | Ion Torrent PGM (IT) |
| coral_IT_low | Coral_13 | 4696544.3 | 2239218 | 197 | Ion Torrent PGM (IT) |
| coral_IT_low | Coral_14 | 4686541.3 | 329583 | 224 | Ion Torrent PGM (IT) |
| kelp_IL_low | CB1SD2_paired | 4717630.3 | 158,628 | 343 | Illumina MiSeq (IL) |
| kelp_IL_low | CB1SD2_2_paired | 4717603.3 | 77,640 | 342 | Illumina MiSeq (IL) |
| kelp_IL_low | CB1SD4_1_paired | 4717607.3 | 174,326 | 322 | Illumina MiSeq (IL) |
| kelp_IL_low | CB1SD4_2_paired | 4717621.3 | 86,594 | 319 | Illumina MiSeq (IL) |
| kelp_IL_low | CB2SD2_1_paired | 4717596.3 | 167,263 | 357 | Illumina MiSeq (IL) |
| kelp_IL_low | CB2SD4_1_paired | 4717597.3 | 118,447 | 353 | Illumina MiSeq (IL) |
| kelp_IL_low | CB2SD4_2_paired | 4717622.3 | 57,526 | 346 | Illumina MiSeq (IL) |
| kelp_IL_low | CB3SD2_1_paired | 4717616.3 | 192,459 | 369 | Illumina MiSeq (IL) |
| kelp_IL_low | CB3SD2_2_paired | 4717612.3 | 98,447 | 368 | Illumina MiSeq (IL) |
| kelp_IL_low | CB3SD4_1_paired | 4717594.3 | 140,443 | 389 | Illumina MiSeq (IL) |
| kelp_IL_low | CB3SD4_2_paired | 4717598.3 | 58,713 | 379 | Illumina MiSeq (IL) |
| kelp_IL_low | CB4DD2_1_paired | 4717601.3 | 105,812 | 337 | Illumina MiSeq (IL) |
| kelp_IL_low | CB4DD2_2_paired.assembled | 4717637.3 | 44,002 | 330 | Illumina MiSeq (IL) |
| kelp_IL_low | CB4DD4_1_paired | 4739056.3 | 105,812 | 337 | Illumina MiSeq (IL) |
| kelp_IL_low | CB4DD4_2_paired | 4739053.3 | 44,002 | 330 | Illumina MiSeq (IL) |
| kelp_IL_low | CB5DD2_1_paired | 4717592.3 | 203,636 | 388 | Illumina MiSeq (IL) |
| kelp_IL_low | CB5DD2_2_paired | 4717617.3 | 100,604 | 385 | Illumina MiSeq (IL) |
| kelp_IL_low | CB5DD4_1_paired | 4717609.3 | 146,367 | 374 | Illumina MiSeq (IL) |
| kelp_IL_low | CB5DD4_2_paired | 4717623.3 | 63,263 | 366 | Illumina MiSeq (IL) |
| kelp_IL_low | CB6DD2_1_paired.assembled | 4717638.3 | 150,586 | 382 | Illumina MiSeq (IL) |
| kelp_IL_low | CB6DD2_2_paired | 4717608.3 | 77,289 | 382 | Illumina MiSeq (IL) |
| kelp_IL_low | CB6DD4_1_paired | 4717593.3 | 151,432 | 327 | Illumina MiSeq (IL) |
| kelp_IL_low | CB6DD4_2_paired | 4717610.3 | 70,410 | 326 | Illumina MiSeq (IL) |
| kelp_IL_low | HB1SD2_1_paired | 4717600.3 | 280,698 | 307 | Illumina MiSeq (IL) |
| kelp_IL_low | HB1SD2_2_paired.assembled | 4717627.3 | 135,366 | 308 | Illumina MiSeq (IL) |
| kelp_IL_low | HB1SD4_1_paired | 4717591.3 | 292.39 | 329 | Illumina MiSeq (IL) |
| kelp_IL_low | HB1SD4_2_paired | 4717634.3 | 122,466 | 327 | Illumina MiSeq (IL) |
| kelp_IL_low | HB2SD2_1_paired | 4717599.3 | 278,341 | 315 | Illumina MiSeq (IL) |
| kelp_IL_low | HB2SD2_2_paired | 4717624.3 | 132,661 | 315 | Illumina MiSeq (IL) |
| kelp_IL_low | HB2SD4_1_paired.assembled | 4717640.3 | 397,414 | 190 | Illumina MiSeq (IL) |
| kelp_IL_low | HB2SD4_2_paired.assembled | 4717629.3 | 161,585 | 197 | Illumina MiSeq (IL) |

(table continues)

**Supplementary Table 1. (continued)**

| kelp_IL_low | HB3SD2_1_paired | 4717613.3 | 276,086 | 326 | Illumina MiSeq (IL) |
| --- | --- | --- | --- | --- | --- |
| kelp_IL_low | HB3SD2_2_paired | 4717628.3 | 127,292 | 324 | Illumina MiSeq (IL) |
| kelp_IL_low | HB3SD4_1_paired | 4717626.3 | 237,873 | 232 | Illumina MiSeq (IL) |
| kelp_IL_low | HB3SD4_2_paired | 4717611.3 | 103,713 | 239 | Illumina MiSeq (IL) |
| kelp_IL_low | HB4DD2_1_paired | 4717620.3 | 131,010 | 335 | Illumina MiSeq (IL) |
| kelp_IL_low | HB4DD2_2_paired.assembled | 4717633.3 | 81,503 | 339 | Illumina MiSeq (IL) |
| kelp_IL_low | HB4DD4_1_paired | 4717605.3 | 200,807 | 299 | Illumina MiSeq (IL) |
| kelp_IL_low | HB4DD4_2_paired.assembled | 4717636.3 | 126,656 | 304 | Illumina MiSeq (IL) |
| kelp_IL_low | HB5DD2_1_paired | 4717618.3 | 161,968 | 349 | Illumina MiSeq (IL) |
| kelp_IL_low | HB5DD2_2_paired | 4717632.3 | 97,647 | 351 | Illumina MiSeq (IL) |
| kelp_IL_low | HB5DD4_1_paired | 4717619.3 | 18,706 | 301 | Illumina MiSeq (IL) |
| kelp_IL_low | HB5DD4_2_paired | 4717604.3 | 11,181 | 310 | Illumina MiSeq (IL) |
| kelp_IL_low | HB6DD2_1_paired | 4739055.3 | 243,878 | 364 | Illumina MiSeq (IL) |
| kelp_IL_low | HB6DD2_2_paired | 4739054.3 | 150,485 | 369 | Illumina MiSeq (IL) |
| kelp_IL_low | HB6DD4_1_paired | 4717614.3 | 17,190 | 310 | Illumina MiSeq (IL) |
| kelp_IL_low | HB6DD4_2_paired | 4717625.3 | 10,272 | 318 | Illumina MiSeq (IL) |
| kelp_IL_low | T03_1_paired | 4717595.3 | 101,824 | 338 | Illumina MiSeq (IL) |
| kelp_IL_low | T03_2_paired | 4717602.3 | 61,488 | 339 | Illumina MiSeq (IL) |
| kelp_IL_low | T04_1_paired.assembled | 4717635.3 | 145,805 | 332 | Illumina MiSeq (IL) |
| kelp_IL_low | T04_2_paired | 4717606.3 | 85,309 | 333 | Illumina MiSeq (IL) |
| kelp_IT_high | PtLomaKelpBenthos12.13.2013 | 4618718.3 | 441,367 | 116 | Ion Torrent PGM (IT) |
| kelp_IT_high | PtLomaKelpBenthos06.27.2013 | 4618708.3 | 54,250 | 195 | Ion Torrent PGM (IT) |
| kelp_IT_high | PtLomaKelpBenthos07.26.2013 | 4618706.3 | 41,827 | 181 | Ion Torrent PGM (IT) |
| kelp_IT_high | PtLomaKelpBenthos07.26.2013 | 4618707.3 | 928,654 | 178 | Ion Torrent PGM (IT) |
| kelp_IT_high | PtLomaKelpBenthos08.20.2013 | 4618713.3 | 40,686 | 167 | Ion Torrent PGM (IT) |
| kelp_IT_high | PtLomaKelpBenthos08.20.2013 | 4618714.3 | 26,760 | 160 | Ion Torrent PGM (IT) |
| kelp_IT_high | PtLomaKelpSurface12.13.2013 | 4618712.3 | 261,763 | 123 | Ion Torrent PGM (IT) |
| kelp_IT_high | PtLomaKelpSurface06.27.2013 | 4618704.3 | 421,855 | 183 | Ion Torrent PGM (IT) |
| kelp_IT_high | PtLomaKelpSurface07.11.2013 | 4618705.3 | 473,213 | 163 | Ion Torrent PGM (IT) |
| kelp_IT_high | PtLomaKelpSurface07.26.2013 | 4618702.3 | 1,641,943 | 183 | Ion Torrent PGM (IT) |
| kelp_IT_high | PtLomaKelpSurface08.20.2013 | 4618717.3 | 89,395 | 193 | Ion Torrent PGM (IT) |
| kelp_IT_high | PtLomaWaterBenthos12.13.2013 | 4618715.3 | 304,675 | 138 | Ion Torrent PGM (IT) |

(table continues)

**Supplementary Table 1. (continued)**

| kelp_IT_high | PtLomaWaterBenthos06.27.2013 | 4618703.3 | 1,546,559 | 187 | Ion Torrent PGM (IT) |
| --- | --- | --- | --- | --- | --- |
| kelp_IT_high | PtLomaKelpBenthos07.11.2013 | 4618701.3 | 308,503 | 179 | Ion Torrent PGM (IT) |
| kelp_IT_high | PtLomaWaterBenthos07.11.2013 | 4618709.3 | 340,805 | 176 | Ion Torrent PGM (IT) |
| kelp_IT_high | PtLomaWaterBenthos08.20.2013 | 4618716.3 | 65,406 | 196 | Ion Torrent PGM (IT) |
| kelp_IT_high | PtLomaWaterSurface12.13.2013 | 4618720.3 | 151,460 | 173 | Ion Torrent PGM (IT) |
| kelp_IT_high | PtLomaWaterSurface06.27.2013 | 4618696.3 | 329,803 | 171 | Ion Torrent PGM (IT) |
| kelp_IT_high | PtLomaWaterSurface06.27.2013 | 4618697.3 | 116,679 | 172 | Ion Torrent PGM (IT) |
| kelp_IT_high | PtLomaWaterSurface07.26.2013 | 4618698.3 | 106,263 | 187 | Ion Torrent PGM (IT) |
| kelp_IT_high | PtLomaWaterSurface07.26.2013 | 4618699.3 | 1,495,075 | 195 | Ion Torrent PGM (IT) |
| kelp_IT_high | PtLomaWaterSurface07.26.2013 | 4618700.3 | 528,395 | 167 | Ion Torrent PGM (IT) |
| kelp_IT_high | PtLomaWaterSurface08.20.2013 | 4618710.3 | 54,616 | 186 | Ion Torrent PGM (IT) |
