# Supplementary material for: Optimizing and evaluating the reconstruction of Metagenome-assembled microbial genomes
Source: BMC Genomics. 2017 Nov 28;18:915. doi: 10.1186/s12864-017-4294-1 (PMC5706307; doi:10.1186/s12864-017-4294-1)
Supplement: Supplementary file 2 — Microbial diversity in the 4 microbiome projects. Representation of microbial diversity using, (a) genus richness, (b) genus evenness, (c) Shannon diversity, and (d) Simpson diversity of the four projects, which are represented on the x axis. The box represents 50% of the data ranges around the median. The outliers for each case are represented as black dots. (DOCX 167 kb) [file 12864_2017_4294_MOESM2_ESM.docx]

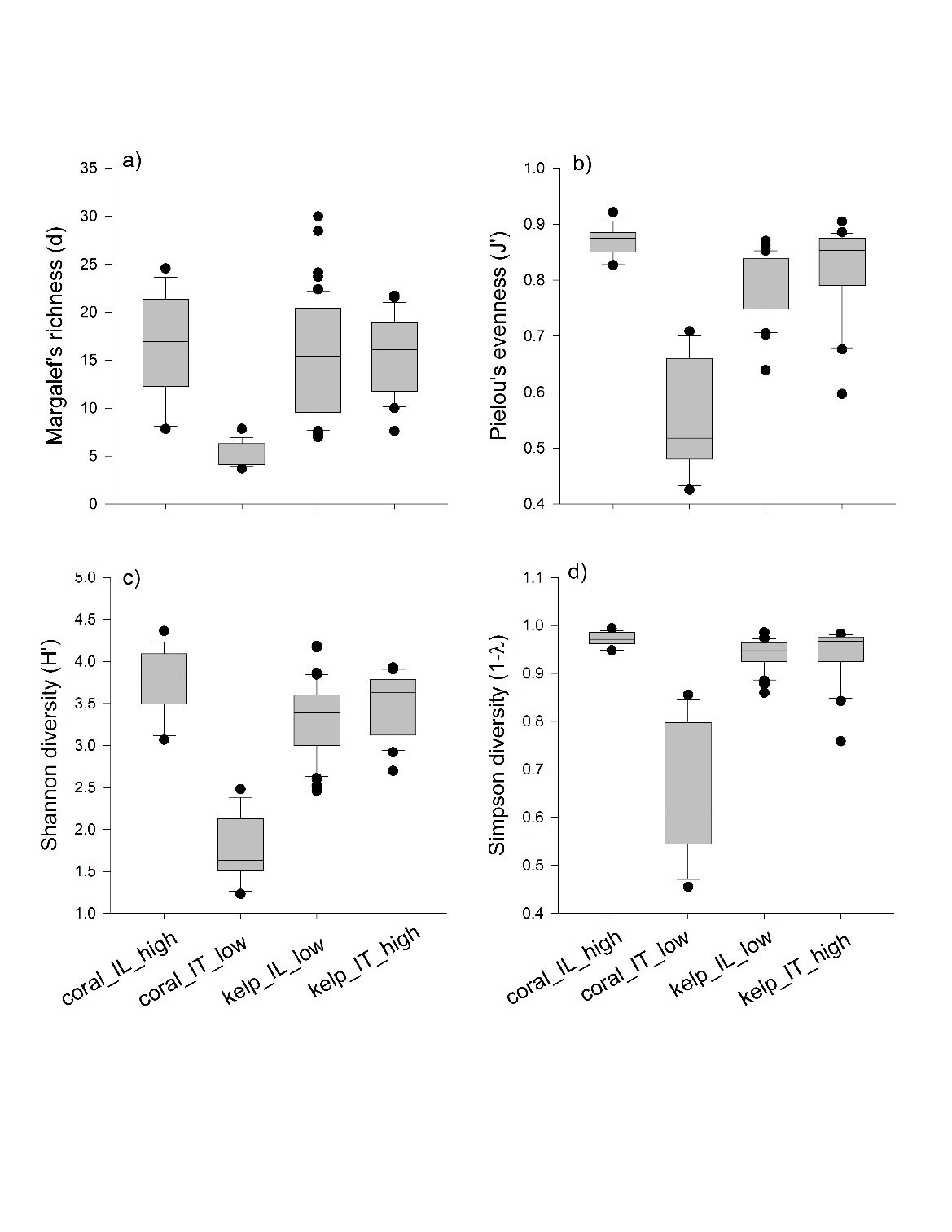


Supplementary Figure 1. Representation of microbial diversity using, (a) genus richness, (b) genus evenness, (c) Shannon diversity, and (d) Simpson diversity of the four projects, which are represented on the x axis. The box represents 50 % of the data ranges around the median. The outliers for each case are represented as black dots.
