# Supplementary material for: Optimizing and evaluating the reconstruction of Metagenome-assembled microbial genomes
Source: BMC Genomics. 2017 Nov 28;18:915. doi: 10.1186/s12864-017-4294-1 (PMC5706307; doi:10.1186/s12864-017-4294-1)
Supplement: Supplementary file 3 — Post hoc Tukey HSD test results for diversity analysis. Post hoc Tukey HSD test results for Shannon, Simpson, Richness and Evenness for the four projects. (DOCX 14 kb) [file 12864_2017_4294_MOESM3_ESM.docx]

Supplementary Table 2. *Post hoc* Tukey HSD test results for Shannon, Simpson, Richness and Evenness for the four projects.

| Tukey-HSD test for Shannon Diversity against the projects | |  |  |  |  |
| --- | --- | --- | --- | --- | --- |
| **Combinations of interaction terms between assembly, project and assembly:project** | | lower limit | upper limit | p value adjusted |  |
| coral_IT_low | coral_IL_high | -2.33 | -1.57 | 0.00 |  |
| kelp_IL_low | coral_IL_high | -0.73 | -0.13 | 0.00 |  |
| kelp_IT_high | coral_IL_high | -0.61 | 0.09 | 0.21 |  |
| kelp_IL_low | coral_IT_low | 1.21 | 1.84 | 0.00 |  |
| kelp_IT_high | coral_IT_low | 1.34 | 2.05 | 0.00 |  |
| kelp_IT_high | kelp_IL_low | -0.10 | 0.44 | 0.37 |  |
|  |  |  |  |  |  |
|  |  |  |  |  |  |
| Tukey-HSD test for Simpson Diversity against the projects | |  |  |  |  |
| **Combinations of interaction terms between assembly, project and assembly:project** | | lower limit | upper limit | p value adjusted |  |
| coral_IT_low | coral_IL_high | -0.37 | -0.26 | 0.00 |  |
| kelp_IL_low | coral_IL_high | -0.08 | 0.02 | 0.31 |  |
| kelp_IT_high | coral_IL_high | -0.08 | 0.02 | 0.47 |  |
| kelp_IL_low | coral_IT_low | 0.24 | 0.33 | 0.00 |  |
| kelp_IT_high | coral_IT_low | 0.23 | 0.34 | 0.00 |  |
| kelp_IT_high | kelp_IL_low | -0.04 | 0.04 | 1.00 |  |
|  |  |  |  |  |  |
| Tukey-HSD test for Richness against the projects | |  |  |  |  |
| **Combinations of interaction terms between assembly, project and assembly:project** | | lower limit | upper limit | p value adjusted |  |
| coral_IT_low | coral_IL_high | -16.14 | -6.81 | 0.00 |  |
| kelp_IL_low | coral_IL_high | -5.13 | 2.30 | 0.75 |  |
| kelp_IT_high | coral_IL_high | -5.43 | 3.10 | 0.89 |  |
| kelp_IL_low | coral_IT_low | 6.25 | 13.87 | 0.00 |  |
| kelp_IT_high | coral_IT_low | 5.97 | 14.66 | 0.00 |  |
| kelp_IT_high | kelp_IL_low | -3.06 | 3.56 | 1.00 |  |
|  |  |  |  |  |  |
| Tukey-HSD test for Evenness against the projects | |  |  |  |  |
| **Combinations of interaction terms between assembly, project and assembly:project** | | lower limit | upper limit | p value adjusted |  |
| coral_IT_low | coral_IL_high | -0.37 | -0.25 | 0.00 |  |
| kelp_IL_low | coral_IL_high | -0.13 | -0.03 | 0.00 |  |
| kelp_IT_high | coral_IL_high | -0.10 | 0.01 | 0.11 |  |
| kelp_IL_low | coral_IT_low | 0.18 | 0.28 | 0.00 |  |
| kelp_IT_high | coral_IT_low | 0.21 | 0.32 | 0.00 |  |
| kelp_IT_high | kelp_IL_low | -0.01 | 0.07 | 0.23 |  |
