# Supplementary material for: Optimizing and evaluating the reconstruction of Metagenome-assembled microbial genomes
Source: BMC Genomics. 2017 Nov 28;18:915. doi: 10.1186/s12864-017-4294-1 (PMC5706307; doi:10.1186/s12864-017-4294-1)
Supplement: Supplementary file 4 — Assembly statistics. QUAST results for the 12 Contigs files assembled using the three assemblers; IDBA, MetaVelvet, SPAdes. (DOCX 16 kb) [file 12864_2017_4294_MOESM4_ESM.docx]

Supplementary Table 3. QUAST results for the 12 Contigs files assembled using the three assemblers; IDBA, MetaVelvet, SPAdes.

| Assembly files | coral_IL_  high_IDBA | coral_IL_  high_velvet | coral_IL_  high_  SPAdes | coral_IT_  low_IDBA | coral_IT_  low_velvet | coral_IT_  low_  SPAdes | kelp_IL_  low_IDBA | kelp_IL_  low_velvet | kelp_IL_  low_  SPAdes | kelp_IT_  low_IDBA | kelp_IT_  low_velvet | kelp_IT_low_SPAdes |
| --- | --- | --- | --- | --- | --- | --- | --- | --- | --- | --- | --- | --- |
| Number of contigs (>=0 bp) | 1187702 | 7172683 | 252654 | 296563 | 220988 | 98605 | 688260 | 3465730 | 188824 | 219184 | 1735743 | 174685 |
| Number of contigs (>= 1000 bp) | 66996 | 11516 | 99373 | 15376 | 19854 | 35282 | 41817 | 11803 | 72609 | 24845 | 1933 | 34824 |
| Number of contigs (>= 5000 bp) | 2885 | 0 | 7031 | 122 | 210 | 2810 | 598 | 4 | 4067 | 1723 | 0 | 2733 |
| Number of contigs (>= 10000 bp) | 630 | 0 | 2055 | 19 | 22 | 439 | 49 | 0 | 1058 | 370 | 0 | 876 |
| Number of contigs (>= 25000 bp) | 46 | 0 | 387 | 4 | 5 | 27 | 4 | 0 | 140 | 9 | 0 | 109 |
| Number of contigs (>= 50000 bp) | 5 | 0 | 73 | 1 | 0 | 5 | 0 | 0 | 18 | 0 | 0 | 9 |
| Total length (>= 0 bp) | 563493075 | 1108606571 | 339701152 | 130213225 | 115638538 | 124792615 | 331803485 | 625272248 | 233886368 | 138562597 | 272583638 | 156812330 |
| Total length (>= 1000 bp) | 132580692 | 14828634 | 240028729 | 23728301 | 31907769 | 86075155 | 70121514 | 15588083 | 156906959 | 55556833 | 2375705 | 85021004 |
| Total length (>= 5000 bp) | 24800612 | 0 | 73464039 | 1020247 | 1583649 | 22343932 | 4230295 | 21862 | 38539883 | 14269642 | 0 | 27497622 |
| Total length (>= 10000 bp) | 9757278 | 0 | 40185335 | 362952 | 397600 | 6725116 | 732734 | 0 | 18406410 | 5148979 | 0 | 14811065 |
| Total length (>= 25000 bp) | 1624457 | 0 | 15591402 | 145594 | 161726 | 1145605 | 122204 | 0 | 5201370 | 285418 | 0 | 3620149 |
| Total length (>= 50000 bp) | 299977 | 0 | 5127936 | 55595 | 0 | 442092 | 0 | 0 | 1147841 | 0 | 0 | 485195 |
| # contigs | 268814 | 138996 | 219623 | 66637 | 71574 | 83105 | 174016 | 119922 | 166749 | 76239 | 30645 | 105395 |
| Largest contig | 67809 | 4911 | 151153 | 55595 | 45475 | 150718 | 38450 | 5802 | 104993 | 38460 | 3650 | 60318 |
| Total length | 266653389 | 95929920 | 334340155 | 58311894 | 67167539 | 118965891 | 158189151 | 85329215 | 230569823 | 90441945 | 20524660 | 132553973 |
| GC (%) | 47.81 | 46.71 | 47.12 | 45.67 | 45.75 | 45.61 | 47.31 | 47.15 | 47.59 | 47.85 | 48.28 | 47.1 |
| N50 | 994 | 661 | 1719 | 873 | 962 | 1898 | 909 | 685 | 1445 | 1322 | 642 | 1466 |
| N75 | 662 | 563 | 940 | 645 | 678 | 932 | 645 | 572 | 891 | 760 | 558 | 783 |
| L50 | 67745 | 54073 | 42234 | 21200 | 21563 | 15572 | 51242 | 45575 | 37461 | 15768 | 12288 | 19135 |
| L75 | 151691 | 93605 | 110448 | 40808 | 42546 | 38547 | 103572 | 79842 | 89608 | 39025 | 20901 | 51185 |
| # N's per 100 kbp | 0 | 0.04 | 0 | 0 | 0 | 0 | 0 | 0 | 0 | 0 | 0 | 0 |
