# Supplementary material for: Optimizing and evaluating the reconstruction of Metagenome-assembled microbial genomes
Source: BMC Genomics. 2017 Nov 28;18:915. doi: 10.1186/s12864-017-4294-1 (PMC5706307; doi:10.1186/s12864-017-4294-1)
Supplement: Supplementary file 5 — Post hoc Tukey HSD test results for contig length. Post hoc Tukey test results comparing contig length of 1000 contigs across assemblers and projects. (DOCX 18 kb) [file 12864_2017_4294_MOESM5_ESM.docx]

Supplementary Table 4. *Post hoc* Tukey test results comparing contig length of 1000 contigs across assemblers and projects.

| Tukey-HSD test for average contig length after 2-way ANOVA | |  |  |  |  |
| --- | --- | --- | --- | --- | --- |
| Assembly variation | lower limit | upper limit | p value adjusted |  |  |
| MetaVelvet | | IDBA | -4.17 | -4.13 | 0.00 |
| SPAdes | IDBA | -0.63 | -0.59 | 0.00 |  |
| SPAdes | MetaVelvet | 3.52 | 3.56 | 0.00 |  |
|  |  |  |  |  |  |
| Project variation | lower limit | upper limit | p value adjusted |  |  |
| coral_IT_low | | coral_IL_high | -2.93 | -2.88 | 0.00 |
| kelp_IL_low | coral_IL_high | -0.41 | -0.36 | 0.00 |  |
| kelp_IT_high | coral_IL_high | -1.79 | -1.74 | 0.00 |  |
| kelp_IL_low | coral_IT_low | 2.49 | 2.54 | 0.00 |  |
| kelp_IT_high | coral_IT_low | 1.11 | 1.17 | 0.00 |  |
| kelp_IT_high | kelp_IL_low | -1.40 | -1.35 | 0.00 |  |
|  |  |  |  |  |  |
| Combinations of interaction terms between assembly, project and assembly:project | lower limit | upper limit | p value adjusted |  |  |
| MetaVelvet: coral_IL_high | | IDBA: coral_IL_high | -3.41 | -3.29 | 0.00 |
| SPAdes: coral_IL_high | IDBA: coral_IL_high | 0.66 | 0.77 | 0.00 |  |
| IDBA: coral_IT_low | IDBA: coral_IL_high | -1.30 | -1.18 | 0.00 |  |
| MetaVelvet: coral_IT_low | IDBA: coral_IL_high | -7.39 | -7.28 | 0.00 |  |
| SPAdes: coral_IT_low | IDBA: coral_IL_high | -2.83 | -2.71 | 0.00 |  |
| IDBA: kelp_IL_low | IDBA: coral_IL_high | -0.80 | -0.69 | 0.00 |  |
| MetaVelvet: kelp_IL_low | IDBA: coral_IL_high | -3.40 | -3.29 | 0.00 |  |
| SPAdes: kelp_IL_low | IDBA: coral_IL_high | 0.23 | 0.35 | 0.00 |  |
| IDBA: kelp_IT_high | IDBA: coral_IL_high | -0.29 | -0.17 | 0.00 |  |
| MetaVelvet: kelp_IT_high | IDBA: coral_IL_high | -4.85 | -4.74 | 0.00 |  |
| SPAdes: kelp_IT_high | IDBA: coral_IL_high | -2.95 | -2.84 | 0.00 |  |
| SPAdes: coral_IL_high | MetaVelvet: coral_IL_high | 4.01 | 4.12 | 0.00 |  |
| IDBA: coral_IT_low | MetaVelvet: coral_IL_high | 2.05 | 2.17 | 0.00 |  |
| MetaVelvet: coral_IT_low | MetaVelvet: coral_IL_high | -4.04 | -3.93 | 0.00 |  |
| SPAdes: coral_IT_low | MetaVelvet: coral_IL_high | 0.52 | 0.64 | 0.00 |  |
| IDBA: kelp_IL_low | MetaVelvet: coral_IL_high | 2.55 | 2.66 | 0.00 |  |
| MetaVelvet: kelp_IL_low | MetaVelvet: coral_IL_high | -0.05 | 0.06 | 1.00 |  |
| SPAdes: kelp_IL_low | MetaVelvet: coral_IL_high | 3.58 | 3.70 | 0.00 |  |
| IDBA: kelp_IT_high | MetaVelvet: coral_IL_high | 3.06 | 3.17 | 0.00 |  |
| MetaVelvet: kelp_IT_high | MetaVelvet: coral_IL_high | -1.50 | -1.39 | 0.00 |  |

(table continues)

**Supplementary Table 4. (continued)**

| SPAdes: kelp_IT_high | MetaVelvet: coral_IL_high | 0.40 | 0.51 | 0.00 |
| --- | --- | --- | --- | --- |
| IDBA: coral_IT_low | SPAdes: coral_IL_high | -2.01 | -1.90 | 0.00 |
| MetaVelvet: coral_IT_low | SPAdes: coral_IL_high | -8.11 | -7.99 | 0.00 |
| SPAdes: coral_IT_low | SPAdes: coral_IL_high | -3.54 | -3.43 | 0.00 |
| IDBA: kelp_IL_low | SPAdes: coral_IL_high | -1.52 | -1.40 | 0.00 |
| MetaVelvet: kelp_IL_low | SPAdes: coral_IL_high | -4.12 | -4.00 | 0.00 |
| SPAdes: kelp_IL_low | SPAdes: coral_IL_high | -0.48 | -0.37 | 0.00 |
| IDBA: kelp_IT_high | SPAdes: coral_IL_high | -1.00 | -0.89 | 0.00 |
| MetaVelvet: kelp_IT_high | SPAdes: coral_IL_high | -5.57 | -5.45 | 0.00 |
| SPAdes: kelp_IT_high | SPAdes: coral_IL_high | -3.66 | -3.55 | 0.00 |
| MetaVelvet: coral_IT_low | IDBA: coral_IT_low | -6.15 | -6.04 | 0.00 |
| SPAdes: coral_IT_low | IDBA: coral_IT_low | -1.59 | -1.47 | 0.00 |
| IDBA: kelp_IL_low | IDBA: coral_IT_low | 0.44 | 0.55 | 0.00 |
| MetaVelvet: kelp_IL_low | IDBA: coral_IT_low | -2.16 | -2.05 | 0.00 |
| SPAdes: kelp_IL_low | IDBA: coral_IT_low | 1.47 | 1.59 | 0.00 |
| IDBA: kelp_IT_high | IDBA: coral_IT_low | 0.95 | 1.06 | 0.00 |
| MetaVelvet: kelp_IT_high | IDBA: coral_IT_low | -3.61 | -3.50 | 0.00 |
| SPAdes: kelp_IT_high | IDBA: coral_IT_low | -1.71 | -1.60 | 0.00 |
| SPAdes: coral_IT_low | MetaVelvet: coral_IT_low | 4.51 | 4.62 | 0.00 |
| IDBA: kelp_IL_low | MetaVelvet: coral_IT_low | 6.53 | 6.65 | 0.00 |
| MetaVelvet: kelp_IL_low | MetaVelvet: coral_IT_low | 3.93 | 4.05 | 0.00 |
| SPAdes: kelp_IL_low | MetaVelvet: coral_IT_low | 7.57 | 7.68 | 0.00 |
| IDBA: kelp_IT_high | MetaVelvet: coral_IT_low | 7.05 | 7.16 | 0.00 |
| MetaVelvet: kelp_IT_high | MetaVelvet: coral_IT_low | 2.48 | 2.60 | 0.00 |
| SPAdes: kelp_IT_high | MetaVelvet: coral_IT_low | 4.38 | 4.50 | 0.00 |
| IDBA: kelp_IL_low | SPAdes: coral_IT_low | 1.97 | 2.08 | 0.00 |
| MetaVelvet: kelp_IL_low | SPAdes: coral_IT_low | -0.63 | -0.52 | 0.00 |
| SPAdes: kelp_IL_low | SPAdes: coral_IT_low | 3.00 | 3.12 | 0.00 |
| IDBA: kelp_IT_high | SPAdes: coral_IT_low | 2.48 | 2.60 | 0.00 |
| MetaVelvet: kelp_IT_high | SPAdes: coral_IT_low | -2.08 | -1.97 | 0.00 |
| SPAdes: kelp_IT_high | SPAdes: coral_IT_low | -0.18 | -0.07 | 0.00 |
| MetaVelvet: kelp_IL_low | IDBA: kelp_IL_low | -2.66 | -2.54 | 0.00 |
| SPAdes: kelp_IL_low | IDBA: kelp_IL_low | 0.98 | 1.09 | 0.00 |
| IDBA: kelp_IT_high | IDBA: kelp_IL_low | 0.46 | 0.57 | 0.00 |
| MetaVelvet: kelp_IT_high | IDBA: kelp_IL_low | -4.11 | -3.99 | 0.00 |
| SPAdes: kelp_IT_high | IDBA: kelp_IL_low | -2.21 | -2.09 | 0.00 |
| SPAdes: kelp_IL_low | MetaVelvet: kelp_IL_low | 3.58 | 3.69 | 0.00 |
| IDBA: kelp_IT_high | MetaVelvet: kelp_IL_low | 3.06 | 3.17 | 0.00 |
| MetaVelvet: kelp_IT_high | MetaVelvet: kelp_IL_low | -1.51 | -1.39 | 0.00 |
| SPAdes: kelp_IT_high | MetaVelvet: kelp_IL_low | 0.39 | 0.51 | 0.00 |

(table continues)

**Supplementary Table 4. (continued)**

| IDBA: kelp_IT_high | SPAdes: kelp_IL_low | -0.58 | -0.46 | 0.00 |
| --- | --- | --- | --- | --- |
| MetaVelvet: kelp_IT_high | SPAdes: kelp_IL_low | -5.14 | -5.03 | 0.00 |
| SPAdes: kelp_IT_high | SPAdes: kelp_IL_low | -3.24 | -3.13 | 0.00 |
| MetaVelvet: kelp_IT_high | IDBA: kelp_IT_high | -4.62 | -4.51 | 0.00 |
| SPAdes: kelp_IT_high | IDBA: kelp_IT_high | -2.72 | -2.61 | 0.00 |
| SPAdes: kelp_IT_high | MetaVelvet: kelp_IT_high | 1.85 | 1.96 | 0.00 |
