# Supplementary material for: Optimizing and evaluating the reconstruction of Metagenome-assembled microbial genomes
Source: BMC Genomics. 2017 Nov 28;18:915. doi: 10.1186/s12864-017-4294-1 (PMC5706307; doi:10.1186/s12864-017-4294-1)
Supplement: Supplementary file 6 — Post hoc Tukey HSD test results for mean reads assembled. Post hoc Tukey test results for the mean reads assembled (%) of 1000 contigs across assemblers and projects. (DOCX 18 kb) [file 12864_2017_4294_MOESM6_ESM.docx]

Supplementary Table 5. *Post hoc* Tukey test results for the mean reads assembled (%) of 1000 contigs across assemblers and projects.

| Tukey-HSD test for reads_assembled_1000 after 2-way ANOVA | |  |  |  |  |  |
| --- | --- | --- | --- | --- | --- | --- |
| Assembly variation | lower limit | upper limit | p value adjusted |  |  |  |
| MetaVelvet | | IDBA | -3.30 | -3.22 | 0.00 | |
| SPAdes | IDBA | 0.33 | 0.40 | 0.00 |  |  |
| SPAdes | MetaVelvet | 3.59 | 3.66 | 0.00 |  |  |
|  |  |  |  |  |  |  |
| Project variation | lower limit | upper limit | p value adjusted |  |  |  |
| coral_IT_low | | coral_IL_high | 0.71 | 0.81 | 0.00 | |
| kelp_IL_low | coral_IL_high | 0.70 | 0.80 | 0.00 |  |  |
| kelp_IT_high | coral_IL_high | -1.25 | -1.15 | 0.00 |  |  |
| kelp_IL_low | coral_IT_low | -0.06 | 0.04 | 0.98 |  |  |
| kelp_IT_high | coral_IT_low | -2.01 | -1.91 | 0.00 |  |  |
| kelp_IT_high | kelp_IL_low | -2.00 | -1.90 | 0.00 |  |  |
|  |  |  |  |  |  |  |
| Combinations of interaction terms between assembly, project and assembly:project | lower limit | upper limit | p value adjusted |  |  |  |
| MetaVelvet: coral_IL_high | | IDBA: coral_IL_high | -5.07 | -4.85 | 0.00 | |
| SPAdes: coral_IL_high | IDBA: coral_IL_high | 0.66 | 0.87 | 0.00 |  |  |
| IDBA: coral_IT_low | IDBA: coral_IL_high | -2.87 | -2.66 | 0.00 |  |  |
| MetaVelvet: coral_IT_low | IDBA: coral_IL_high | -1.28 | -1.06 | 0.00 |  |  |
| SPAdes: coral_IT_low | IDBA: coral_IL_high | 1.91 | 2.13 | 0.00 |  |  |
| IDBA: kelp_IL_low | IDBA: coral_IL_high | 1.51 | 1.72 | 0.00 |  |  |
| MetaVelvet: kelp_IL_low | IDBA: coral_IL_high | -4.22 | -4.00 | 0.00 |  |  |
| SPAdes: kelp_IL_low | IDBA: coral_IL_high | 0.44 | 0.66 | 0.00 |  |  |
| IDBA: kelp_IT_high | IDBA: coral_IL_high | -0.38 | -0.16 | 0.00 |  |  |
| MetaVelvet: kelp_IT_high | IDBA: coral_IL_high | -4.34 | -4.12 | 0.00 |  |  |
| SPAdes: kelp_IT_high | IDBA: coral_IL_high | -3.41 | -3.19 | 0.00 |  |  |
| SPAdes: coral_IL_high | MetaVelvet: coral_IL_high | 5.62 | 5.83 | 0.00 |  |  |
| IDBA: coral_IT_low | MetaVelvet: coral_IL_high | 2.09 | 2.30 | 0.00 |  |  |

(table continues)

**Supplementary Table 5. (continued)**

| MetaVelvet: coral_IT_low | MetaVelvet: coral_IL_high | 3.68 | 3.90 | 0.00 |
| --- | --- | --- | --- | --- |
| SPAdes: coral_IT_low | MetaVelvet: coral_IL_high | 6.87 | 7.09 | 0.00 |
| IDBA: kelp_IL_low | MetaVelvet: coral_IL_high | 6.47 | 6.68 | 0.00 |
| MetaVelvet: kelp_IL_low | MetaVelvet: coral_IL_high | 0.74 | 0.96 | 0.00 |
| SPAdes: kelp_IL_low | MetaVelvet: coral_IL_high | 5.41 | 5.62 | 0.00 |
| IDBA: kelp_IT_high | MetaVelvet: coral_IL_high | 4.58 | 4.80 | 0.00 |
| MetaVelvet: kelp_IT_high | MetaVelvet: coral_IL_high | 0.62 | 0.84 | 0.00 |
| SPAdes: kelp_IT_high | MetaVelvet: coral_IL_high | 1.55 | 1.77 | 0.00 |
| IDBA: coral_IT_low | SPAdes: coral_IL_high | -3.64 | -3.42 | 0.00 |
| MetaVelvet: coral_IT_low | SPAdes: coral_IL_high | -2.04 | -1.83 | 0.00 |
| SPAdes: coral_IT_low | SPAdes: coral_IL_high | 1.14 | 1.36 | 0.00 |
| IDBA: kelp_IL_low | SPAdes: coral_IL_high | 0.74 | 0.96 | 0.00 |
| MetaVelvet: kelp_IL_low | SPAdes: coral_IL_high | -4.98 | -4.77 | 0.00 |
| SPAdes: kelp_IL_low | SPAdes: coral_IL_high | -0.32 | -0.10 | 0.00 |
| IDBA: kelp_IT_high | SPAdes: coral_IL_high | -1.14 | -0.93 | 0.00 |
| MetaVelvet: kelp_IT_high | SPAdes: coral_IL_high | -5.10 | -4.89 | 0.00 |
| SPAdes: kelp_IT_high | SPAdes: coral_IL_high | -4.17 | -3.96 | 0.00 |
| MetaVelvet: coral_IT_low | IDBA: coral_IT_low | 1.49 | 1.71 | 0.00 |
| SPAdes: coral_IT_low | IDBA: coral_IT_low | 4.67 | 4.89 | 0.00 |
| IDBA: kelp_IL_low | IDBA: coral_IT_low | 4.27 | 4.49 | 0.00 |
| MetaVelvet: kelp_IL_low | IDBA: coral_IT_low | -1.45 | -1.23 | 0.00 |
| SPAdes: kelp_IL_low | IDBA: coral_IT_low | 3.21 | 3.43 | 0.00 |
| IDBA: kelp_IT_high | IDBA: coral_IT_low | 2.39 | 2.60 | 0.00 |
| MetaVelvet: kelp_IT_high | IDBA: coral_IT_low | -1.57 | -1.35 | 0.00 |
| SPAdes: kelp_IT_high | IDBA: coral_IT_low | -0.64 | -0.43 | 0.00 |
| SPAdes: coral_IT_low | MetaVelvet: coral_IT_low | 3.08 | 3.29 | 0.00 |
| IDBA: kelp_IL_low | MetaVelvet: coral_IT_low | 2.68 | 2.89 | 0.00 |

(table continues)

**Supplementary Table 5. (continued)**

| MetaVelvet: kelp_IL_low | MetaVelvet: coral_IT_low | -3.05 | -2.83 | 0.00 |
| --- | --- | --- | --- | --- |
| SPAdes: kelp_IL_low | MetaVelvet: coral_IT_low | 1.61 | 1.83 | 0.00 |
| IDBA: kelp_IT_high | MetaVelvet: coral_IT_low | 0.79 | 1.01 | 0.00 |
| MetaVelvet: kelp_IT_high | MetaVelvet: coral_IT_low | -3.17 | -2.95 | 0.00 |
| SPAdes: kelp_IT_high | MetaVelvet: coral_IT_low | -2.24 | -2.02 | 0.00 |
| IDBA: kelp_IL_low | SPAdes: coral_IT_low | -0.51 | -0.29 | 0.00 |
| MetaVelvet: kelp_IL_low | SPAdes: coral_IT_low | -6.23 | -6.02 | 0.00 |
| SPAdes: kelp_IL_low | SPAdes: coral_IT_low | -1.57 | -1.36 | 0.00 |
| IDBA: kelp_IT_high | SPAdes: coral_IT_low | -2.39 | -2.18 | 0.00 |
| MetaVelvet: kelp_IT_high | SPAdes: coral_IT_low | -6.35 | -6.14 | 0.00 |
| SPAdes: kelp_IT_high | SPAdes: coral_IT_low | -5.42 | -5.21 | 0.00 |
| MetaVelvet: kelp_IL_low | IDBA: kelp_IL_low | -5.83 | -5.62 | 0.00 |
| SPAdes: kelp_IL_low | IDBA: kelp_IL_low | -1.17 | -0.95 | 0.00 |
| IDBA: kelp_IT_high | IDBA: kelp_IL_low | -1.99 | -1.78 | 0.00 |
| MetaVelvet: kelp_IT_high | IDBA: kelp_IL_low | -5.95 | -5.74 | 0.00 |
| SPAdes: kelp_IT_high | IDBA: kelp_IL_low | -5.02 | -4.81 | 0.00 |
| SPAdes: kelp_IL_low | MetaVelvet: kelp_IL_low | 4.55 | 4.77 | 0.00 |
| IDBA: kelp_IT_high | MetaVelvet: kelp_IL_low | 3.73 | 3.95 | 0.00 |
| MetaVelvet: kelp_IT_high | MetaVelvet: kelp_IL_low | -0.23 | -0.01 | 0.02 |
| SPAdes: kelp_IT_high | MetaVelvet: kelp_IL_low | 0.70 | 0.92 | 0.00 |
| IDBA: kelp_IT_high | SPAdes: kelp_IL_low | -0.93 | -0.71 | 0.00 |
| MetaVelvet: kelp_IT_high | SPAdes: kelp_IL_low | -4.89 | -4.67 | 0.00 |
| SPAdes: kelp_IT_high | SPAdes: kelp_IL_low | -3.96 | -3.74 | 0.00 |
| MetaVelvet: kelp_IT_high | IDBA: kelp_IT_high | -4.07 | -3.85 | 0.00 |
| SPAdes: kelp_IT_high | IDBA: kelp_IT_high | -3.14 | -2.92 | 0.00 |
| SPAdes: kelp_IT_high | MetaVelvet: kelp_IT_high | 0.82 | 1.04 | 0.00 |
