# Supplementary material for: Optimizing and evaluating the reconstruction of Metagenome-assembled microbial genomes
Source: BMC Genomics. 2017 Nov 28;18:915. doi: 10.1186/s12864-017-4294-1 (PMC5706307; doi:10.1186/s12864-017-4294-1)
Supplement: Supplementary file 7 — Assembly evaluation parameters. List of all the assembly evaluation parameters. (DOCX 16 kb) [file 12864_2017_4294_MOESM7_ESM.docx]

Supplementary Table 6. List of all the assembly evaluation parameters.

| Contig Length  1000 contigs | IDBA | IDBA standard error | MetaVelvet | MetaVelvet standard error | SPAdes | SPAdes standard error |
| --- | --- | --- | --- | --- | --- | --- |
| coral_IL_high | 11203 | 2646 | 369 | 113 | 22728 | 5797 |
| coral_IT_low | 3359 | 1094 | 8 | 7 | 697 | 299 |
| kelp_IL_low | 5365 | 1088 | 395 | 110 | 14957 | 3660 |
| kelp_IT_high | 8742 | 1719 | 122 | 44 | 638 | 51 |
|  |  |  |  |  |  |  |
| Reads assembled (%) 1000 contigs | IDBA | IDBA standard error | MetaVelvet | MetaVelvet standard error | SPAdes | SPAdes standard error |
| coral_IL_high | 0.002 | 9.21E-05 | 0.000 | 8.20E-07 | 0.005 | 0.000227 |
| coral_IT_low | 0.000 | 1.58E-05 | 0.001 | 1.72E-05 | 0.015 | 0.000352 |
| kelp_IL_low | 0.015 | 0.000236 | 0.000 | 9.63E-07 | 0.008 | 0.000811 |
| kelp_IT_high | 0.002 | 8.42E-05 | 0.000 | 9.41E-07 | 0.000 | 1.12E-06 |
|  |  |  |  |  |  |  |
| Number of contigs | IDBA | MetaVelvet | SPAdes |  |  |  |
| coral_IL_high | 268814 | 138996 | 219623 |  |  |  |
| coral_IT_low | 66637 | 71574 | 83105 |  |  |  |
| kelp_IL_low | 174016 | 120057 | 166749 |  |  |  |
| kelp_IT_high | 76239 | 30645 | 105395 |  |  |  |
|  |  |  |  |  |  |  |
| N-50 length | IDBA | MetaVelvet | SPAdes |  |  |  |
| coral_IL_high | 909 | 686 | 1445 |  |  |  |
| coral_IT_low | 1322 | 642 | 1466 |  |  |  |
| kelp_IL_low | 994 | 661 | 1719 |  |  |  |
| kelp_IT_high | 873 | 962 | 1898 |  |  |  |
|  |  |  |  |  |  |  |
| Reads assembled  all contigs | IDBA | MetaVelvet | SPAdes |  |  |  |
| coral_IL_high | 19.24 | 9.6 | 22.04 |  |  |  |
| coral_IT_low | 4.06 | 0.48 | 9.51 |  |  |  |
| kelp_IL_low | 27.61 | 20.83 | 28.69 |  |  |  |
| kelp_IT_high | 16.38 | 7.3 | 18.36 |  |  |  |
|  |  |  |  |  |  |  |
| Richness | IDBA | MetaVelvet | SPAdes | Reads |  |  |
| coral_IL_high | 19.76 | 16.29 | 17.81 | 15.30 |  |  |
| coral_IT_low | 10.21 | 7.82 | 8.03 | 6.95 |  |  |
| kelp_IL_low | 24.75 | 21.93 | 22.37 | 17.59 |  |  |
| kelp_IT_high | 21.50 | 22.37 | 21.71 | 23.67 |  |  |

(table continues)

**Supplementary Table 6. (continued)**

| Evenness | IDBA | MetaVelvet | SPAdes | Reads |  |  |
| --- | --- | --- | --- | --- | --- | --- |
| coral_IL_high | 0.92 | 0.91 | 0.89 | 0.79 |  |  |
| coral_IT_low | 0.67 | 0.68 | 0.60 | 0.63 |  |  |
| kelp_IL_low | 0.84 | 0.85 | 0.82 | 0.83 |  |  |
| kelp_IT_high | 0.86 | 0.87 | 0.87 | 0.86 |  |  |
