# Supplementary material for: Optimizing and evaluating the reconstruction of Metagenome-assembled microbial genomes
Source: BMC Genomics. 2017 Nov 28;18:915. doi: 10.1186/s12864-017-4294-1 (PMC5706307; doi:10.1186/s12864-017-4294-1)
Supplement: Supplementary file 8 — Binning tool evaluation parameters. List of the parameters for the GroopM and MetaBat extracted bins. (DOCX 51 kb) [file 12864_2017_4294_MOESM8_ESM.docx]

Supplementary Table 7. List of the parameters for the GroopM and MetaBat extracted bins.

| Project | Bins | Binning tool | GC | Number of contigs | GC content variation | Species Richness | Completeness |
| --- | --- | --- | --- | --- | --- | --- | --- |
| coral_IL_high | 1 | MetaBat | 37.73 | 57 | 1.80 | 5.21 | 5.07 |
| coral_IL_high | 2 | MetaBat | 41.47 | 103 | 3.51 | 4.13 | 0 |
| coral_IL_high | 3 | MetaBat | 48.20 | 283 | 2.72 | 7.82 | 22.14 |
| coral_IL_high | 4 | MetaBat | 38.00 | 73 | 3.94 | 6.73 | 0 |
| coral_IL_high | 5 | MetaBat | 51.89 | 78 | 3.86 | 5.86 | 1.1 |
| coral_IL_high | 6 | MetaBat | 54.40 | 150 | 0.62 | 3.69 | 6.27 |
| coral_IL_high | 7 | MetaBat | 45.60 | 133 | 3.92 | 3.04 | 10.34 |
| coral_IL_high | 8 | MetaBat | 32.91 | 216 | 5.37 | 6.95 | 5.36 |
| coral_IL_high | 9 | MetaBat | 37.28 | 848 | 2.96 | 13.90 | 29.37 |
| coral_IL_high | 10 | MetaBat | 60.24 | 1687 | 4.76 | 2.39 | 81.43 |
| coral_IL_high | 11 | MetaBat | 34.31 | 1868 | 2.69 | 13.25 | 93.1 |
| coral_IL_high | 12 | MetaBat | 44.33 | 1892 | 3.55 | 1.74 | 70.4 |
| coral_IL_high | 13 | MetaBat | 53.66 | 769 | 3.55 | 3.26 | 100 |
| coral_IL_high | 14 | MetaBat | 51.94 | 1215 | 1.42 | 10.21 | 99.14 |
| coral_IL_high | 15 | MetaBat | 40.31 | 490 | 6.25 | 7.38 | 100 |
| coral_IL_high | 16 | MetaBat | 32.81 | 1165 | 5.13 | 8.03 | 15.86 |
| coral_IL_high | 17 | MetaBat | 59.24 | 684 | 0.92 | 4.13 | 92.63 |
| coral_IL_high | 18 | MetaBat | 41.11 | 174 | 6.75 | 6.95 | 11.26 |
| coral_IL_high | 19 | MetaBat | 31.54 | 194 | 3.25 | 2.82 | 4.67 |
| coral_IL_high | 20 | MetaBat | 53.26 | 295 | 2.85 | 2.39 | 95.69 |
| coral_IL_high | 21 | MetaBat | 49.58 | 211 | 6.47 | 3.69 | 97.41 |
| coral_IL_high | 22 | MetaBat | 55.80 | 654 | 2.62 | 14.77 | 74.14 |
| coral_IL_high | 23 | MetaBat | 39.59 | 938 | 5.44 | 7.17 | 0 |
| coral_IL_high | 24 | MetaBat | 42.07 | 805 | 0.80 | 12.16 | 37.59 |
| coral_IL_high | 25 | MetaBat | 45.90 | 253 | 2.27 | 3.91 | 12.07 |
| coral_IL_high | 26 | MetaBat | 34.57 | 1554 | 3.74 | 12.81 | 87.93 |
| coral_IL_high | 27 | MetaBat | 41.38 | 315 | 3.13 | 13.90 | 34.62 |
| coral_IL_high | 28 | MetaBat | 39.38 | 270 | 5.11 | 9.34 | 52.27 |

(table continues)

**Supplementary Table 7. (continued)**

| coral_IL_high | 29 | MetaBat | 41.55 | 512 | 5.58 | 9.99 | 22.41 |
| --- | --- | --- | --- | --- | --- | --- | --- |
| coral_IL_high | 30 | MetaBat | 40.37 | 256 | 2.11 | 10.42 | 6.74 |
| coral_IL_high | 31 | MetaBat | 58.88 | 787 | 4.03 | 3.26 | 81.55 |
| coral_IL_high | 32 | MetaBat | 42.76 | 726 | 6.54 | 3.69 | 34.35 |
| coral_IL_high | 33 | MetaBat | 53.47 | 474 | 4.84 | 16.50 | 39.8 |
| coral_IL_high | 34 | MetaBat | 41.10 | 726 | 4.11 | 7.82 | 37.08 |
| coral_IL_high | 35 | MetaBat | 49.28 | 326 | 2.21 | 4.56 | 21.5 |
| coral_IL_high | 36 | MetaBat | 56.28 | 489 | 1.73 | 4.13 | 10.29 |
| coral_IL_high | 37 | MetaBat | 59.40 | 879 | 1.74 | 4.99 | 22.53 |
| coral_IL_high | 38 | MetaBat | 59.00 | 186 | 3.65 | 3.04 | 0.78 |
| coral_IL_high | 39 | MetaBat | 41.54 | 200 | 3.30 | 10.42 | 9.09 |
| coral_IL_high | 40 | MetaBat | 53.37 | 445 | 3.35 | 6.08 | 40.13 |
| coral_IL_high | 41 | MetaBat | 56.74 | 825 | 2.75 | 7.38 | 79.67 |
| coral_IL_high | 42 | MetaBat | 52.61 | 270 | 2.31 | 6.73 | 15.36 |
| coral_IL_high | 43 | MetaBat | 54.04 | 215 | 4.15 | 6.08 | 3.45 |
| coral_IL_high | 44 | MetaBat | 36.89 | 240 | 4.66 | 9.99 | 41.36 |
| coral_IL_high | 45 | MetaBat | 38.98 | 728 | 3.29 | 15.20 | 39.58 |
| coral_IL_high | 46 | MetaBat | 56.16 | 701 | 5.18 | 4.56 | 31.66 |
| coral_IL_high | 47 | MetaBat | 38.81 | 611 | 2.20 | 16.94 | 41.37 |
| coral_IL_high | 48 | MetaBat | 61.50 | 284 | 2.76 | 5.21 | 7.05 |
| coral_IL_high | 49 | MetaBat | 51.06 | 3536 | 1.75 | 6.51 | 50.62 |
| coral_IL_high | 50 | MetaBat | 45.07 | 2318 | 4.28 | 2.61 | 81.35 |
| coral_IL_high | 51 | MetaBat | 43.74 | 238 | 4.23 | 5.86 | 18.53 |
| coral_IL_high | 52 | MetaBat | 39.86 | 665 | 2.05 | 7.38 | 36.21 |
| coral_IL_high | 53 | MetaBat | 43.97 | 326 | 4.46 | 4.34 | 44.38 |
| coral_IL_high | 54 | MetaBat | 37.78 | 297 | 4.73 | 9.12 | 93.1 |
| coral_IL_high | 55 | MetaBat | 44.19 | 230 | 1.62 | 6.95 | 7.52 |
| coral_IL_high | 56 | MetaBat | 43.64 | 266 | 4.98 | 4.13 | 39.22 |
| coral_IL_high | 57 | MetaBat | 43.21 | 276 | 4.71 | 7.38 | 12.23 |
| coral_IL_high | 1 | GroopM | 48.11 | 1415 | 1.04 | 13.68 | 44.84 |
| coral_IL_high | 2 | GroopM | 63.78 | 242 | 3.94 | 7.17 | 15.36 |
| coral_IL_high | 3 | GroopM | 39.11 | 186 | 0.44 | 14.98 | 17.79 |
| coral_IL_high | 4 | GroopM | 46.64 | 28 | 4.51 | 6.30 | 0 |
| coral_IL_high | 5 | GroopM | 47.30 | 680 | 1.99 | 14.55 | 16.91 |
| coral_IL_high | 6 | GroopM | 58.43 | 28 | 1.72 | 10.86 | 2.41 |
| coral_IL_high | 7 | GroopM | 57.69 | 11 | 2.99 | 6.95 | 0 |
| coral_IL_high | 8 | GroopM | 53.33 | 195 | 2.80 | 8.69 | 8.07 |
| coral_IL_high | 9 | GroopM | 57.13 | 60 | 5.72 | 14.11 | 2.59 |
| coral_IL_high | 10 | GroopM | 44.58 | 585 | 2.02 | 10.42 | 31.24 |
| coral_IL_high | 11 | GroopM | 31.05 | 70 | 2.38 | 6.51 | 26.14 |

(table continues)

**Supplementary Table 7. (continued)**

| coral_IL_high | 12 | GroopM | 53.32 | 340 | 3.55 | 8.03 | 11.72 |
| --- | --- | --- | --- | --- | --- | --- | --- |
| coral_IL_high | 13 | GroopM | 49.43 | 1486 | 0.87 | 13.25 | 43.81 |
| coral_IL_high | 14 | GroopM | 42.30 | 17 | 5.49 | 5.21 | 0.16 |
| coral_IL_high | 15 | GroopM | 35.64 | 76 | 1.58 | 7.38 | 40.52 |
| coral_IL_high | 16 | GroopM | 41.24 | 36 | 1.41 | 4.56 | 0 |
| coral_IL_high | 17 | GroopM | 25.07 | 50 | 6.21 | 2.82 | 0.78 |
| coral_IL_high | 18 | GroopM | 43.36 | 201 | 4.67 | 9.12 | 57.58 |
| coral_IL_high | 19 | GroopM | 53.03 | 29 | 1.03 | 13.03 | 0 |
| coral_IL_high | 20 | GroopM | 35.65 | 74 | 4.05 | 8.03 | 1.72 |
| coral_IL_high | 21 | GroopM | 35.08 | 221 | 4.49 | 13.68 | 17.87 |
| coral_IL_high | 22 | GroopM | 45.45 | 441 | 3.25 | 11.94 | 15.2 |
| coral_IL_high | 23 | GroopM | 49.78 | 356 | 2.21 | 10.86 | 5.49 |
| coral_IL_high | 24 | GroopM | 50.96 | 166 | 5.40 | 7.38 | 1.72 |
| coral_IL_high | 25 | GroopM | 42.92 | 2828 | 2.68 | 19.54 | 100 |
| coral_IL_high | 26 | GroopM | 55.97 | 715 | 3.02 | 11.51 | 24.28 |
| coral_IL_high | 27 | GroopM | 53.37 | 415 | 0.80 | 10.86 | 18.81 |
| coral_IL_high | 28 | GroopM | 23.24 | 27 | 1.34 | 2.82 | 0 |
| coral_IL_high | 29 | GroopM | 59.93 | 545 | 1.63 | 7.60 | 45.69 |
| coral_IL_high | 30 | GroopM | 44.11 | 319 | 0.84 | 4.13 | 45.61 |
| coral_IL_high | 31 | GroopM | 52.51 | 329 | 0.48 | 5.21 | 37.07 |
| coral_IL_high | 32 | GroopM | 47.92 | 20 | 2.81 | 8.90 | 0 |
| coral_IL_high | 33 | GroopM | 35.08 | 3860 | 2.01 | 12.38 | 100 |
| coral_IL_high | 34 | GroopM | 54.66 | 1368 | 1.45 | 9.34 | 32.82 |
| coral_IL_high | 35 | GroopM | 42.64 | 924 | 2.71 | 12.59 | 45.17 |
| coral_IL_high | 36 | GroopM | 52.62 | 9329 | 0.89 | 9.34 | 100 |
| coral_IL_high | 37 | GroopM | 39.91 | 379 | 2.08 | 9.34 | 58.7 |
| coral_IL_high | 38 | GroopM | 34.14 | 526 | 0.47 | 11.51 | 43.34 |
| coral_IL_high | 39 | GroopM | 47.80 | 46 | 3.48 | 6.51 | 3.61 |
| coral_IL_high | 40 | GroopM | 43.46 | 74 | 1.79 | 12.38 | 10.34 |
| coral_IL_high | 41 | GroopM | 31.52 | 1290 | 3.13 | 8.90 | 100 |
| coral_IL_high | 42 | GroopM | 47.95 | 4840 | 0.77 | 12.16 | 69.22 |
| coral_IL_high | 43 | GroopM | 44.93 | 32 | 1.16 | 5.86 | 0.63 |
| coral_IL_high | 44 | GroopM | 29.97 | 352 | 0.78 | 4.56 | 74.14 |
| coral_IL_high | 45 | GroopM | 37.94 | 87 | 2.74 | 9.12 | 2.04 |
| coral_IL_high | 46 | GroopM | 57.19 | 4179 | 1.33 | 4.99 | 100 |
| coral_IL_high | 47 | GroopM | 45.43 | 1588 | 3.81 | 4.34 | 67.87 |
| coral_IL_high | 48 | GroopM | 40.06 | 642 | 1.81 | 8.69 | 35.44 |
| coral_IL_high | 49 | GroopM | 54.17 | 449 | 0.35 | 7.82 | 11.47 |
| coral_IL_high | 50 | GroopM | 46.68 | 42 | 0.59 | 7.82 | 5.17 |
| coral_IL_high | 51 | GroopM | 43.32 | 20 | 0.81 | 6.95 | 1.88 |

(table continues)

**Supplementary Table 7. (continued)**

| coral_IL_high | 52 | GroopM | 44.03 | 164 | 2.68 | 8.90 | 26.19 |
| --- | --- | --- | --- | --- | --- | --- | --- |
| coral_IL_high | 53 | GroopM | 53.75 | 672 | 0.83 | 9.12 | 8.73 |
| coral_IL_high | 54 | GroopM | 41.12 | 181 | 0.69 | 13.46 | 11.41 |
| coral_IL_high | 55 | GroopM | 37.04 | 46 | 1.44 | 7.82 | 16.16 |
| coral_IL_high | 56 | GroopM | 37.85 | 2 | 1.31 | 5.43 | 6.1 |
| coral_IL_high | 57 | GroopM | 32.21 | 201 | 1.08 | 8.69 | 12.93 |
| coral_IL_high | 58 | GroopM | 21.02 | 140 | 2.10 | 2.17 | 5.36 |
| coral_IL_high | 59 | GroopM | 59.20 | 53 | 1.20 | 9.12 | 0 |
| coral_IL_high | 60 | GroopM | 58.43 | 28 | 2.25 | 4.13 | 10.58 |
| coral_IL_high | 61 | GroopM | 38.46 | 1675 | 1.19 | 7.38 | 5.33 |
| coral_IL_high | 62 | GroopM | 38.99 | 28 | 3.36 | 9.55 | 3.45 |
| coral_IL_high | 63 | GroopM | 52.99 | 554 | 2.28 | 8.47 | 18.05 |
| coral_IL_high | 64 | GroopM | 34.25 | 99 | 0.70 | 5.21 | 46.55 |
| coral_IL_high | 65 | GroopM | 40.08 | 133 | 1.48 | 12.38 | 16.71 |
| coral_IL_high | 66 | GroopM | 53.88 | 204 | 5.34 | 3.69 | 18.01 |
| coral_IL_high | 67 | GroopM | 43.92 | 843 | 3.48 | 15.20 | 37.55 |
| coral_IL_high | 68 | GroopM | 37.68 | 392 | 3.58 | 15.85 | 19.36 |
| coral_IL_high | 69 | GroopM | 58.08 | 283 | 0.85 | 8.90 | 50 |
| coral_IL_high | 70 | GroopM | 65.81 | 146 | 0.92 | 4.56 | 9.64 |
| coral_IL_high | 71 | GroopM | 44.70 | 176 | 2.27 | 8.03 | 9.91 |
| coral_IL_high | 72 | GroopM | 31.12 | 265 | 4.07 | 9.77 | 15.47 |
| coral_IL_high | 73 | GroopM | 51.64 | 1014 | 4.83 | 11.94 | 40.75 |
| coral_IL_high | 74 | GroopM | 47.41 | 308 | 1.48 | 15.63 | 3.13 |
| coral_IT_low | 1 | MetaBat | 37.78 | 149 | 1.76 | 7.17 | 9.12 |
| coral_IT_low | 2 | MetaBat | 30.53 | 217 | 2.70 | 3.47 | 19.98 |
| coral_IT_low | 3 | MetaBat | 41.78 | 171 | 1.32 | 6.08 | 9.64 |
| coral_IT_low | 4 | MetaBat | 41.60 | 802 | 1.24 | 1.52 | 19.47 |
| coral_IT_low | 5 | MetaBat | 46.02 | 163 | 0.89 | 2.82 | 21.55 |
| coral_IT_low | 6 | MetaBat | 41.41 | 364 | 2.16 | 4.13 | 12.85 |
| coral_IT_low | 7 | MetaBat | 48.04 | 510 | 2.32 | 4.99 | 8.93 |
| coral_IT_low | 8 | MetaBat | 60.33 | 216 | 2.12 | 3.26 | 3.92 |
| coral_IT_low | 9 | MetaBat | 48.93 | 261 | 1.40 | 4.78 | 11.21 |
| coral_IT_low | 10 | MetaBat | 45.98 | 888 | 2.77 | 4.99 | 43.76 |
| coral_IT_low | 11 | MetaBat | 28.82 | 907 | 1.83 | 3.69 | 90.44 |
| coral_IT_low | 12 | MetaBat | 44.53 | 329 | 0.66 | 4.78 | 23.18 |
| coral_IT_low | 13 | MetaBat | 46.09 | 10 | 4.70 | 3.04 | 0.31 |
| coral_IT_low | 14 | MetaBat | 39.25 | 51 | 0.95 | 2.61 | 0 |
| coral_IT_low | 15 | MetaBat | 45.07 | 2176 | 0.9253428 | 3.47 | 77.04 |
| coral_IT_low | 16 | MetaBat | 40.94 | 1563 | 0.95 | 2.82 | 63.11 |
| coral_IT_low | 17 | MetaBat | 51.94 | 410 | 2.16 | 3.91 | 31.03 |

(table continues)

**Supplementary Table 7 (continued)**

| coral_IT_low | 1 | GroopM | 42.34 | 8057 | 3.27 | 11.94 | 49.91 |
| --- | --- | --- | --- | --- | --- | --- | --- |
| coral_IT_low | 2 | GroopM | 41.31 | 1512 | 2.93 | 9.34 | 13.75 |
| coral_IT_low | 3 | GroopM | 52.10 | 7675 | 1.79 | 6.73 | 81.74 |
| coral_IT_low | 4 | GroopM | 29.98 | 1113 | 2.84 | 3.26 | 33.39 |
| coral_IT_low | 5 | GroopM | 55.47 | 6018 | 2.89 | 8.03 | 69.35 |
| coral_IT_low | 6 | GroopM | 42.29 | 2022 | 1.13 | 6.08 | 11.38 |
| coral_IT_low | 7 | GroopM | 27.12 | 272 | 1.15 | 3.47 | 0.16 |
| coral_IT_low | 8 | GroopM | 62.24 | 193 | 1.36 | 3.69 | 3.45 |
| coral_IT_low | 9 | GroopM | 45.48 | 2441 | 2.42 | 3.69 | 97.93 |
| coral_IT_low | 10 | GroopM | 45.33 | 1832 | 1.40 | 8.47 | 26.28 |
| coral_IT_low | 11 | GroopM | 28.52 | 182 | 1.73 | 2.39 | 3.79 |
| coral_IT_low | 12 | GroopM | 45.61 | 9194 | 1.57 | 6.73 | 63.57 |
| coral_IT_low | 13 | GroopM | 27.99 | 927 | 3.22 | 2.82 | 86.13 |
| coral_IT_low | 14 | GroopM | 37.96 | 1159 | 2.89 | 5.86 | 40.1 |
| coral_IT_low | 15 | GroopM | 50.63 | 998 | 1.76 | 6.08 | 14.42 |
| coral_IT_low | 16 | GroopM | 25.87 | 62 | 1.36 | 2.17 | 0 |
| coral_IT_low | 17 | GroopM | 60.22 | 458 | 1.70 | 4.99 | 8.78 |
| coral_IT_low | 18 | GroopM | 45.27 | 4177 | 1.25 | 7.60 | 55.62 |
| coral_IT_low | 19 | GroopM | 45.47 | 1019 | 4.39 | 4.13 | 20.86 |
| coral_IT_low | 20 | GroopM | 49.62 | 1148 | 1.69 | 11.29 | 13.01 |
| coral_IT_low | 21 | GroopM | 46.33 | 3501 | 3.92 | 5.65 | 11.36 |
| coral_IT_low | 22 | GroopM | 52.07 | 1971 | 3.00 | 11.29 | 15.23 |
| coral_IT_low | 23 | GroopM | 44.83 | 11532 | 3.06 | 6.08 | 88.22 |
| coral_IT_low | 24 | GroopM | 42.80 | 1704 | 3.07 | 12.38 | 13.79 |
| coral_IT_low | 25 | GroopM | 46.34 | 1019 | 1.57 | 8.03 | 17.87 |
| coral_IT_low | 26 | GroopM | 59.33 | 744 | 3.82 | 6.30 | 8.7 |
| coral_IT_low | 27 | GroopM | 41.23 | 204 | 2.67 | 7.38 | 4.49 |
| coral_IT_low | 28 | GroopM | 55.74 | 578 | 2.49 | 6.30 | 13.24 |
| coral_IT_low | 29 | GroopM | 32.87 | 363 | 2.91 | 6.30 | 5.64 |
| coral_IT_low | 30 | GroopM | 45.33 | 5153 | 1.85 | 9.12 | 53.15 |
| coral_IT_low | 31 | GroopM | 41.78 | 4238 | 2.22 | 5.21 | 51.91 |
| kelp_IL_low | 1 | MetaBat | 60.66 | 487 | 0.51 | 4.56 | 27.27 |
| kelp_IL_low | 2 | MetaBat | 42.82 | 93 | 0.80 | 2.17 | 14.66 |
| kelp_IL_low | 3 | MetaBat | 45.62 | 128 | 0.48 | 3.47 | 6.9 |
| kelp_IL_low | 4 | MetaBat | 45.26 | 53 | 1.21 | 4.34 | 3.45 |
| kelp_IL_low | 5 | MetaBat | 62.82 | 1046 | 1.09 | 4.78 | 90.7 |
| kelp_IL_low | 6 | MetaBat | 62.35 | 181 | 0.97 | 6.73 | 10.34 |
| kelp_IL_low | 7 | MetaBat | 43.15 | 375 | 0.66 | 5.21 | 34.48 |
| kelp_IL_low | 8 | MetaBat | 54.46 | 65 | 1.46 | 2.61 | 8.62 |
| kelp_IL_low | 9 | MetaBat | 60.14 | 441 | 1.05 | 4.56 | 92.71 |

(table continues)

**Supplementary Table 7. (continued)**

| kelp_IL_low | 10 | MetaBat | 40.20 | 2305 | 0.98 | 3.69 | 80.8 |
| --- | --- | --- | --- | --- | --- | --- | --- |
| kelp_IL_low | 11 | MetaBat | 44.16 | 1440 | 0.62 | 5.65 | 80.33 |
| kelp_IL_low | 12 | MetaBat | 44.42 | 227 | 0.53 | 2.17 | 26.02 |
| kelp_IL_low | 13 | MetaBat | 56.88 | 74 | 1.14 | 2.61 | 18.1 |
| kelp_IL_low | 14 | MetaBat | 40.28 | 718 | 1.12 | 5.21 | 87.93 |
| kelp_IL_low | 15 | MetaBat | 33.14 | 528 | 0.86 | 3.91 | 42.48 |
| kelp_IL_low | 16 | MetaBat | 37.77 | 80 | 1.04 | 4.34 | 0 |
| kelp_IL_low | 17 | MetaBat | 52.90 | 1123 | 3.44 | 2.61 | 98.2 |
| kelp_IL_low | 1 | GroopM | 43.14 | 172 | 4.17 | 14.11 | 16.71 |
| kelp_IL_low | 2 | GroopM | 46.71 | 428 | 4.69 | 16.94 | 26.72 |
| kelp_IL_low | 3 | GroopM | 51.46 | 73 | 4.86 | 14.55 | 3.97 |
| kelp_IL_low | 4 | GroopM | 50.85 | 759 | 1.24 | 17.59 | 30.41 |
| kelp_IL_low | 5 | GroopM | 64.60 | 55 | 1.57 | 6.08 | 1.72 |
| kelp_IL_low | 6 | GroopM | 46.20 | 156 | 2.41 | 9.99 | 12.07 |
| kelp_IL_low | 7 | GroopM | 43.99 | 175 | 0.91 | 15.85 | 13.24 |
| kelp_IL_low | 8 | GroopM | 53.48 | 393 | 5.21 | 2.82 | 44.2 |
| kelp_IL_low | 9 | GroopM | 47.16 | 315 | 4.11 | 18.02 | 13.5 |
| kelp_IL_low | 10 | GroopM | 51.36 | 650 | 4.82 | 18.46 | 37.05 |
| kelp_IL_low | 11 | GroopM | 50.85 | 541 | 4.15 | 22.15 | 27.93 |
| kelp_IL_low | 12 | GroopM | 54.56 | 173 | 3.99 | 9.55 | 6.33 |
| kelp_IL_low | 13 | GroopM | 53.70 | 630 | 3.41 | 16.94 | 23.15 |
| kelp_IL_low | 14 | GroopM | 48.64 | 984 | 1.67 | 12.38 | 35.74 |
| kelp_IL_low | 15 | GroopM | 63.88 | 178 | 0.70 | 7.17 | 4.23 |
| kelp_IL_low | 16 | GroopM | 51.00 | 29 | 3.02 | 6.51 | 0 |
| kelp_IL_low | 17 | GroopM | 45.21 | 352 | 1.34 | 17.81 | 14.66 |
| kelp_IL_low | 18 | GroopM | 38.31 | 46 | 4.21 | 8.90 | 1.72 |
| kelp_IL_low | 19 | GroopM | 55.47 | 54 | 6.06 | 11.51 | 3.45 |
| kelp_IL_low | 20 | GroopM | 49.23 | 71 | 0.67 | 16.94 | 2.59 |
| kelp_IL_low | 21 | GroopM | 66.46 | 21 | 3.57 | 4.56 | 3.45 |
| kelp_IL_low | 22 | GroopM | 42.92 | 397 | 4.82 | 16.50 | 19.01 |
| kelp_IL_low | 23 | GroopM | 52.60 | 311 | 1.97 | 21.50 | 15.6 |
| kelp_IL_low | 24 | GroopM | 39.03 | 97 | 2.27 | 8.03 | 6.11 |
| kelp_IL_low | 25 | GroopM | 38.57 | 164 | 4.17 | 12.81 | 4.31 |
| kelp_IL_low | 26 | GroopM | 53.19 | 250 | 3.65 | 17.59 | 6.11 |
| kelp_IL_low | 27 | GroopM | 45.98 | 117 | 3.09 | 10.64 | 7.52 |
| kelp_IL_low | 28 | GroopM | 44.76 | 324 | 1.01 | 16.50 | 15.02 |
| kelp_IL_low | 29 | GroopM | 64.38 | 42 | 1.77 | 4.99 | 1.47 |
| kelp_IL_low | 30 | GroopM | 39.58 | 921 | 1.84 | 6.51 | 70.69 |
| kelp_IL_low | 31 | GroopM | 43.47 | 154 | 1.33 | 13.03 | 9.56 |
| kelp_IL_low | 32 | GroopM | 53.15 | 130 | 1.73 | 7.17 | 8.62 |

(table continues)

**Supplementary Table 7. (continued)**

| kelp_IL_low | 33 | GroopM | 36.52 | 37 | 4.73 | 9.34 | 0 |
| --- | --- | --- | --- | --- | --- | --- | --- |
| kelp_IL_low | 34 | GroopM | 51.65 | 385 | 4.52 | 15.20 | 20.3 |
| kelp_IL_low | 35 | GroopM | 51.82 | 144 | 2.90 | 16.29 | 11.5 |
| kelp_IL_low | 36 | GroopM | 38.63 | 58 | 4.14 | 6.73 | 0.86 |
| kelp_IL_low | 37 | GroopM | 44.43 | 116 | 3.05 | 16.72 | 9.09 |
| kelp_IL_low | 38 | GroopM | 45.67 | 916 | 2.43 | 7.82 | 54.31 |
| kelp_IL_low | 39 | GroopM | 41.68 | 79 | 4.39 | 11.94 | 4.78 |
| kelp_IL_low | 40 | GroopM | 51.39 | 136 | 1.80 | 15.20 | 3.76 |
| kelp_IL_low | 41 | GroopM | 52.67 | 55 | 2.26 | 11.51 | 1.72 |
| kelp_IL_low | 42 | GroopM | 61.62 | 86 | 3.50 | 8.47 | 1.25 |
| kelp_IL_low | 43 | GroopM | 42.94 | 262 | 5.15 | 16.50 | 13.4 |
| kelp_IL_low | 44 | GroopM | 51.74 | 95 | 4.09 | 13.90 | 3.45 |
| kelp_IL_low | 45 | GroopM | 51.71 | 65 | 3.35 | 12.81 | 4.86 |
| kelp_IL_low | 46 | GroopM | 53.35 | 58 | 1.13 | 11.94 | 6.9 |
| kelp_IL_low | 47 | GroopM | 58.76 | 67 | 0.99 | 8.47 | 6.03 |
| kelp_IL_low | 48 | GroopM | 63.73 | 157 | 2.53 | 7.60 | 18.5 |
| kelp_IL_low | 49 | GroopM | 38.49 | 153 | 1.11 | 13.90 | 12.07 |
| kelp_IL_low | 50 | GroopM | 63.50 | 18 | 3.35 | 4.78 | 0 |
| kelp_IL_low | 51 | GroopM | 36.36 | 21 | 3.08 | 6.51 | 0 |
| kelp_IL_low | 52 | GroopM | 43.69 | 169 | 4.22 | 14.55 | 5.02 |
| kelp_IL_low | 53 | GroopM | 56.51 | 133 | 4.23 | 14.33 | 4.31 |
| kelp_IL_low | 54 | GroopM | 55.42 | 41 | 2.15 | 9.77 | 3.45 |
| kelp_IL_low | 55 | GroopM | 42.68 | 2175 | 2.43 | 11.07 | 9.64 |
| kelp_IL_low | 56 | GroopM | 42.68 | 2175 | 5.23 | 13.03 | 78.34 |
| kelp_IL_low | 57 | GroopM | 52.73 | 83 | 1.46 | 14.33 | 1.72 |
| kelp_IL_low | 58 | GroopM | 64.17 | 50 | 1.44 | 6.08 | 0 |
| kelp_IL_low | 59 | GroopM | 64.03 | 111 | 2.84 | 5.86 | 3.45 |
| kelp_IL_low | 60 | GroopM | 40.37 | 321 | 2.92 | 9.55 | 5.17 |
| kelp_IL_low | 61 | GroopM | 56.11 | 66 | 1.17 | 9.99 | 3.45 |
| kelp_IL_low | 62 | GroopM | 31.75 | 21 | 1.49 | 5.43 | 0 |
| kelp_IL_low | 63 | GroopM | 32.90 | 48 | 5.68 | 6.73 | 1.02 |
| kelp_IL_low | 64 | GroopM | 49.33 | 117 | 3.84 | 18.46 | 4.47 |
| kelp_IL_low | 65 | GroopM | 53.42 | 88 | 3.28 | 11.94 | 10.34 |
| kelp_IL_low | 66 | GroopM | 53.27 | 36 | 0.98 | 5.65 | 0 |
| kelp_IL_low | 67 | GroopM | 64.09 | 93 | 0.66 | 5.21 | 6.5 |
| kelp_IL_low | 68 | GroopM | 51.28 | 109 | 2.08 | 3.69 | 21.15 |
| kelp_IL_low | 69 | GroopM | 39.18 | 263 | 4.28 | 8.90 | 6.96 |
| kelp_IL_low | 70 | GroopM | 45.92 | 649 | 2.10 | 16.50 | 41.61 |
| kelp_IL_low | 71 | GroopM | 41.85 | 965 | 4.00 | 11.73 | 42.16 |
| kelp_IL_low | 72 | GroopM | 49.90 | 647 | 2.40 | 9.99 | 52.12 |

(table continues)

**Supplementary Table 7. (continued)**

| kelp_IL_low | 73 | GroopM | 37.95 | 1151 | 2.19 | 12.38 | 46.93 |
| --- | --- | --- | --- | --- | --- | --- | --- |
| kelp_IL_low | 74 | GroopM | 39.51 | 148 | 2.46 | 15.20 | 3.45 |
| kelp_IL_low | 75 | GroopM | 57.49 | 32 | 3.25 | 9.55 | 0 |
| kelp_IL_low | 76 | GroopM | 40.65 | 97 | 1.15 | 18.02 | 0.47 |
| kelp_IL_low | 77 | GroopM | 62.94 | 95 | 0.42 | 8.47 | 2.59 |
| kelp_IL_low | 78 | GroopM | 54.53 | 82 | 1.66 | 2.82 | 5.17 |
| kelp_IL_low | 79 | GroopM | 41.73 | 211 | 1.51 | 11.94 | 10.03 |
| kelp_IL_low | 80 | GroopM | 64.58 | 92 | 1.21 | 8.90 | 8.62 |
| kelp_IL_low | 81 | GroopM | 53.07 | 226 | 2.51 | 4.99 | 30.71 |
| kelp_IL_low | 82 | GroopM | 40.18 | 233 | 1.52 | 14.77 | 18.18 |
| kelp_IL_low | 83 | GroopM | 63.00 | 208 | 0.76 | 7.38 | 11.44 |
| kelp_IL_low | 84 | GroopM | 63.43 | 46 | 0.76 | 5.65 | 3.92 |
| kelp_IL_low | 85 | GroopM | 52.67 | 72 | 1.85 | 4.99 | 5.17 |
| kelp_IL_low | 86 | GroopM | 38.66 | 371 | 1.37 | 8.69 | 23.82 |
| kelp_IL_low | 87 | GroopM | 62.58 | 554 | 1.59 | 3.91 | 29.15 |
| kelp_IL_low | 88 | GroopM | 44.36 | 559 | 5.38 | 9.77 | 31.66 |
| kelp_IL_low | 89 | GroopM | 47.17 | 61 | 1.20 | 15.42 | 0 |
| kelp_IL_low | 90 | GroopM | 54.65 | 88 | 1.34 | 4.13 | 12.15 |
| kelp_IL_low | 91 | GroopM | 42.14 | 1645 | 3.72 | 6.08 | 88.01 |
| kelp_IL_low | 92 | GroopM | 48.37 | 887 | 1.28 | 13.68 | 34.33 |
| kelp_IL_low | 93 | GroopM | 63.95 | 250 | 2.72 | 9.34 | 20.45 |
| kelp_IL_low | 94 | GroopM | 44.59 | 854 | 0.66 | 14.98 | 51.71 |
| kelp_IL_low | 95 | GroopM | 58.80 | 23 | 1.91 | 6.08 | 3.45 |
| kelp_IL_low | 96 | GroopM | 42.95 | 562 | 1.13 | 13.03 | 25.28 |
| kelp_IL_low | 97 | GroopM | 64.11 | 63 | 3.17 | 5.65 | 3.45 |
| kelp_IL_low | 98 | GroopM | 51.33 | 286 | 2.76 | 4.99 | 33.7 |
| kelp_IL_low | 99 | GroopM | 50.00 | 238 | 4.37 | 6.08 | 11.44 |
| kelp_IL_low | 100 | GroopM | 46.58 | 353 | 4.27 | 14.33 | 29.28 |
| kelp_IL_low | 101 | GroopM | 54.47 | 618 | 2.75 | 9.55 | 28.21 |
| kelp_IL_low | 102 | GroopM | 44.77 | 721 | 1.78 | 14.11 | 31.61 |
| kelp_IL_low | 103 | GroopM | 42.52 | 180 | 1.40 | 9.55 | 11.13 |
| kelp_IL_low | 104 | GroopM | 60.27 | 408 | 3.64 | 4.34 | 89.89 |
| kelp_IL_low | 105 | GroopM | 52.96 | 172 | 4.46 | 4.99 | 24.8 |
| kelp_IL_low | 106 | GroopM | 49.64 | 416 | 1.07 | 16.94 | 36.42 |
| kelp_IL_low | 107 | GroopM | 62.67 | 85 | 0.61 | 6.73 | 2.59 |
| kelp_IL_low | 108 | GroopM | 61.15 | 18 | 1.78 | 6.73 | 0 |
| kelp_IL_low | 109 | GroopM | 39.07 | 298 | 2.54 | 9.55 | 16.3 |
| kelp_IL_low | 110 | GroopM | 40.93 | 51 | 1.30 | 9.12 | 2.76 |
| kelp_IL_low | 111 | GroopM | 62.27 | 616 | 3.29 | 4.56 | 74.29 |
| kelp_IL_low | 112 | GroopM | 58.38 | 238 | 1.18 | 10.64 | 19.44 |

(table continues)

**Supplementary Table 7. (continued)**

| kelp_IL_low | 113 | GroopM | 32.17 | 61 | 1.29 | 4.78 | 6.97 |
| --- | --- | --- | --- | --- | --- | --- | --- |
| kelp_IL_low | 114 | GroopM | 39.98 | 135 | 2.37 | 6.73 | 8.62 |
| kelp_IL_low | 115 | GroopM | 50.83 | 4 | 1.19 | 6.51 | 0 |
| kelp_IL_low | 116 | GroopM | 64.26 | 103 | 1.25 | 5.86 | 3.45 |
| kelp_IL_low | 117 | GroopM | 44.52 | 109 | 1.97 | 6.95 | 11.44 |
| kelp_IT_high | 1 | MetaBat | 51.56 | 848 | 2.97 | 4.99 | 82.45 |
| kelp_IT_high | 2 | MetaBat | 49.02 | 1019 | 3.87 | 5.43 | 85.16 |
| kelp_IT_high | 3 | MetaBat | 41.76 | 658 | 1.92 | 2.82 | 78.68 |
| kelp_IT_high | 4 | MetaBat | 57.08 | 973 | 3.05 | 6.08 | 35.34 |
| kelp_IT_high | 5 | MetaBat | 55.43 | 910 | 4.93 | 2.39 | 79.9 |
| kelp_IT_high | 6 | MetaBat | 58.34 | 659 | 2.75 | 6.51 | 47.15 |
| kelp_IT_high | 7 | MetaBat | 43.28 | 487 | 2.49 | 8.03 | 77.27 |
| kelp_IT_high | 8 | MetaBat | 52.09 | 1224 | 3.58 | 3.69 | 54.55 |
| kelp_IT_high | 9 | MetaBat | 48.46 | 1682 | 3.48 | 9.99 | 73.12 |
| kelp_IT_high | 10 | MetaBat | 48.03 | 280 | 1.95 | 8.25 | 22.71 |
| kelp_IT_high | 11 | MetaBat | 56.11 | 1025 | 3.86 | 6.51 | 39.83 |
| kelp_IT_high | 12 | MetaBat | 48.92 | 1016 | 3.07 | 12.59 | 55.62 |
| kelp_IT_high | 13 | MetaBat | 48.35 | 720 | 4.73 | 8.69 | 37.27 |
| kelp_IT_high | 14 | MetaBat | 34.79 | 500 | 1.21 | 11.94 | 32.68 |
| kelp_IT_high | 15 | MetaBat | 52.30 | 176 | 3.41 | 6.73 | 28.86 |
| kelp_IT_high | 16 | MetaBat | 39.50 | 1011 | 2.24 | 11.94 | 57.94 |
| kelp_IT_high | 17 | MetaBat | 56.98 | 667 | 3.09 | 6.30 | 37.21 |
| kelp_IT_high | 18 | MetaBat | 37.76 | 577 | 3.35 | 6.08 | 97.07 |
| kelp_IT_high | 19 | MetaBat | 51.94 | 525 | 3.20 | 6.08 | 24.25 |
| kelp_IT_high | 20 | MetaBat | 51.98 | 836 | 2.39 | 3.69 | 84.8 |
| kelp_IT_high | 21 | MetaBat | 49.99 | 758 | 3.56 | 2.39 | 68.54 |
| kelp_IT_high | 22 | MetaBat | 47.29 | 865 | 2.96 | 11.73 | 40.49 |
| kelp_IT_high | 23 | MetaBat | 53.06 | 257 | 1.50 | 12.81 | 15.67 |
| kelp_IT_high | 24 | MetaBat | 44.84 | 701 | 0.33 | 4.34 | 92.71 |
| kelp_IT_high | 1 | GroopM | 51.37 | 31 | 1.35 | 8.69 | 0 |
| kelp_IT_high | 2 | GroopM | 51.17 | 1291 | 1.59 | 8.90 | 80.15 |
| kelp_IT_high | 3 | GroopM | 34.46 | 325 | 1.19 | 11.07 | 10.66 |
| kelp_IT_high | 4 | GroopM | 51.41 | 934 | 0.79 | 6.30 | 89.5 |
| kelp_IT_high | 5 | GroopM | 44.78 | 141 | 1.77 | 11.94 | 8.78 |
| kelp_IT_high | 6 | GroopM | 48.79 | 910 | 1.98 | 4.13 | 79.12 |
| kelp_IT_high | 7 | GroopM | 34.42 | 600 | 1.63 | 11.29 | 29.45 |
| kelp_IT_high | 8 | GroopM | 55.45 | 1557 | 3.80 | 6.08 | 81.87 |
| kelp_IT_high | 9 | GroopM | 41.04 | 775 | 0.64 | 13.25 | 64.81 |
| kelp_IT_high | 10 | GroopM | 61.40 | 134 | 0.61 | 6.30 | 3.45 |
| kelp_IT_high | 11 | GroopM | 52.76 | 209 | 1.73 | 3.04 | 17.59 |

(table continues)

**Supplementary Table 7. (continued)**

| kelp_IT_high | 12 | GroopM | 36.18 | 115 | 1.42 | 7.82 | 0 |
| --- | --- | --- | --- | --- | --- | --- | --- |
| kelp_IT_high | 13 | GroopM | 37.47 | 134 | 1.98 | 9.55 | 9.09 |
| kelp_IT_high | 14 | GroopM | 41.51 | 684 | 1.91 | 3.26 | 92.89 |
| kelp_IT_high | 15 | GroopM | 51.88 | 502 | 1.09 | 8.47 | 47.38 |
| kelp_IT_high | 16 | GroopM | 37.90 | 129 | 1.15 | 11.73 | 2.3 |
| kelp_IT_high | 17 | GroopM | 37.48 | 298 | 1.00 | 2.82 | 12.15 |
| kelp_IT_high | 18 | GroopM | 55.19 | 149 | 1.47 | 4.78 | 9.33 |
| kelp_IT_high | 19 | GroopM | 43.04 | 162 | 1.39 | 10.21 | 19.02 |
| kelp_IT_high | 20 | GroopM | 30.97 | 490 | 3.42 | 7.82 | 32.11 |
| kelp_IT_high | 21 | GroopM | 46.51 | 216 | 1.04 | 15.42 | 9.48 |
| kelp_IT_high | 22 | GroopM | 46.98 | 68 | 1.89 | 6.95 | 1.72 |
| kelp_IT_high | 23 | GroopM | 34.62 | 124 | 2.01 | 7.82 | 6.5 |
| kelp_IT_high | 24 | GroopM | 60.72 | 743 | 2.43 | 5.43 | 51.69 |
| kelp_IT_high | 25 | GroopM | 49.36 | 37 | 4.29 | 4.99 | 0 |
| kelp_IT_high | 26 | GroopM | 44.24 | 246 | 1.17 | 12.59 | 22.88 |
| kelp_IT_high | 27 | GroopM | 44.70 | 697 | 2.26 | 4.56 | 86.18 |
| kelp_IT_high | 28 | GroopM | 39.44 | 152 | 1.90 | 10.86 | 4.23 |
| kelp_IT_high | 29 | GroopM | 52.90 | 2544 | 0.91 | 4.99 | 95.98 |
| kelp_IT_high | 30 | GroopM | 42.71 | 57 | 2.38 | 8.25 | 7.13 |
| kelp_IT_high | 31 | GroopM | 56.47 | 148 | 4.12 | 7.38 | 0.34 |
| kelp_IT_high | 32 | GroopM | 42.90 | 314 | 4.13 | 14.55 | 16.14 |
| kelp_IT_high | 33 | GroopM | 45.02 | 318 | 2.72 | 18.02 | 18.03 |
| kelp_IT_high | 34 | GroopM | 44.38 | 28 | 3.64 | 9.99 | 0 |
| kelp_IT_high | 35 | GroopM | 42.04 | 81 | 2.06 | 14.55 | 5.17 |
| kelp_IT_high | 36 | GroopM | 59.48 | 84 | 3.66 | 8.90 | 0 |
| kelp_IT_high | 37 | GroopM | 45.30 | 84 | 0.83 | 14.98 | 3.45 |
| kelp_IT_high | 38 | GroopM | 36.75 | 406 | 1.04 | 5.43 | 80.45 |
| kelp_IT_high | 39 | GroopM | 57.55 | 863 | 2.16 | 2.39 | 33.62 |
