# Supplementary material for: Optimizing and evaluating the reconstruction of Metagenome-assembled microbial genomes
Source: BMC Genomics. 2017 Nov 28;18:915. doi: 10.1186/s12864-017-4294-1 (PMC5706307; doi:10.1186/s12864-017-4294-1)
Supplement: Supplementary file 9 — Comparison on metagenome-assembled genomes. Comparison of the genome parameters of novel metagenome-assembled genome (coral_IL_high Bin 13) against the three closest genomes from the database. (DOCX 13 kb) [file 12864_2017_4294_MOESM9_ESM.docx]

Supplementary Table 8. Comparison of the genome parameters of novel metagenome-assembled genome (coral_IL_high Bin 13) against the three closest genomes from the database.

| Genomes | Novel metagenome-assembled genome | *Parvibaculum lavamentivorans* | Alpha proteobacterium IMCC 14465 | *Pelagibacter ubique* |
| --- | --- | --- | --- | --- |
| GC content (%) | 54 | 62.3 | 47.4 | 26.7 |
| Genome size (Mbp) | 3.96 | 3.91 | 1.90 | 1.31 |
| Number of protein encoding genes | 4342 | 3648 | 1797 | 1354 |
| Number of RNA sequences | 57 | 60 | 36 | 35 |
