# Supplementary material for: Optimizing and evaluating the reconstruction of Metagenome-assembled microbial genomes
Source: BMC Genomics. 2017 Nov 28;18:915. doi: 10.1186/s12864-017-4294-1 (PMC5706307; doi:10.1186/s12864-017-4294-1)
Supplement: Supplementary file 10 — Optimized workflow. Guide to optimized workflow to reconstruct metagenome-assembled genomes. Description of the programs used in this study at each step and the evaluation parameters calculation is provided as step by step workflow. (DOCX 148 kb) [file 12864_2017_4294_MOESM10_ESM.docx]

GUide to Optimized workflow to reconstruct metagenome-assembled genomes


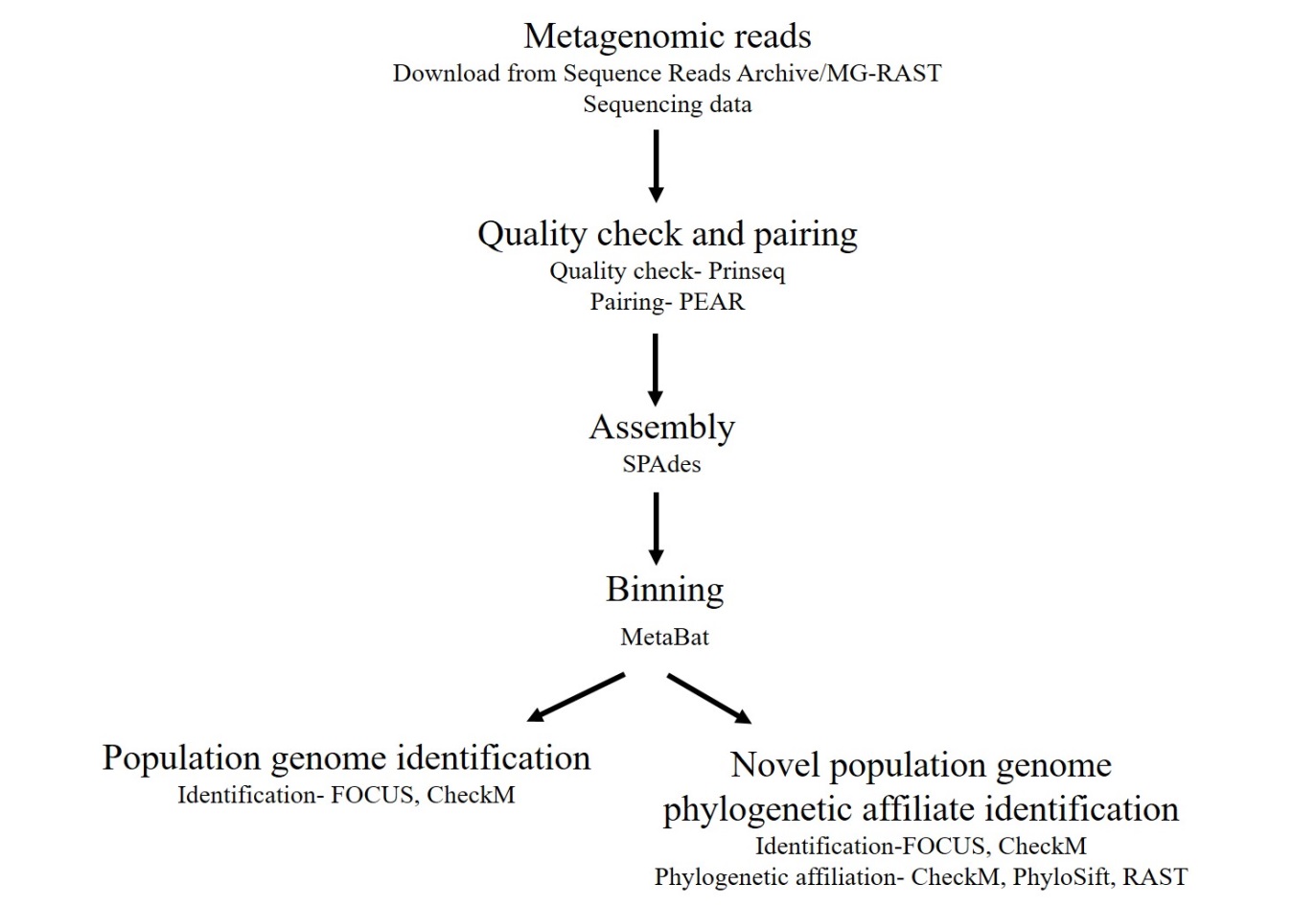


Figure 2. Optimized workflow to reconstruct population genomes from microbiomes, beginning with quality check, assembly, binning and population genome identification. The workflow includes an additional step to identify the reconstructed novel population genomes its phylogenetic neighbors.

Steps to install and run the tools to reconstruct metagenome-assembled genomes from microbiome using the above workflow.

1. Quality check is the first step, where the metagenomes summary statistics including the number of read sequences, minimum length, maximum length, quality scores for fastq sequences, number of sequence duplicates, ambiguous bases (N’s), sequence complexity, tag sequences are calculated. PRINSEQ, PRe-processing and INformation of the SEQuence data was applied for this step to check for quality of sequences and remove duplicates, ambiguous bases, and tag sequences.

**Prinseq** can be downloaded from <https://sourceforge.net/projects/prinseq/files/> to your local computer or online version is available at <http://edwards.sdsu.edu/cgi-bin/prinseq/prinseq.cgi>.

In the paper, I applied the following command to remove duplicate sequences, since the microbiomes was already pre-processed for other projects, and were downloaded from MG-RAST. The first command takes in one input fasta sequence, and removes de- replicates to output the good quality sequences to “filename good” and the filtered sequences to “filename bad”.

*perl* [*prinseq-lite.pl*](http://prinseq-lite.pl/) *-fasta <filename> -derep 12345 --out_format 1 -out_good <filename good> –out_bad < filename bad>*

The below command includes the same command as above but for paired end sequences, the difference being that the forward and reverse reads must be provided as inputs.

*perl* [*prinseq-lite.pl*](http://prinseq-lite.pl/) *-fasta <filename> -fasta2 <filename> -derep 12345 --out_format 1 -out_good “$f”_good*

Finally, all the metagenomes summary statistics was calculated using the command below.

*perl* [*prinseq-lite.p*](http://prinseq-lite.pl/)*l -fastq <good fastq file> -fastq2 <good fastq file> -stats_all*

1. For Illumina paired samples, the forward and reverse reads were paired post quality check step to form longer read sequences. To perform this step, Paired-End reAd merger (PEAR) was applied. PEAR can be downloaded and installed from <https://github.com/xflouris/PEAR>. The input sequences include forward and reverse reads that contain only the good quality sequences, the output from PRINSEQ step.

./pear -f <forward reads.fastq > -r <reverse reads.fastq> -o <output file.fastq>

The output of this tool gives you two files; assembled, and unassembled, along with the summary statistics including the percent of reads that were assembled.

1. Metagenome assembly using SPAdes. This step requires a large amount of memory and may take a while depending on the size of the input. First all the metagenomes (reads) per project were placed in one file which was the input for assembly.

*cat <reads file> <AllReads.fasta>*

SPAdes can be downloaded and installed from <http://spades.bioinf.spbau.ru/release3.10.1/manual.html>. The manual for SPAdes is well explained with a detailed description of all the commands and how they can be applied. The command applied in this study for IonTorrent samples is given below. The input for the command requires the reads file, -s to specify that they are unpaired samples, *--iontorrent* to specify the sequencer type, and –only-assembler was specified to reduce the time and memory required by the program.

*python spades.py  --iontorrent -s <AllReads.fasta> --only-assembler -o <output filename>*

In the case of the paired Illumina reads, the only difference is that no sequencer type was specified in the command. Since the sequences were already paired, the input includes the concatenated paired read file.

*spades.py –s <Allreads.fasta>* *--only-assembler -o <output filename>*

The contigs can be found in the output folder, under the folder with different k-mer size. SPAdes iteratively increases the k-mer sizes to improve assembly to the highest k-mer size of 127.

1. Binning was performed using the Contig Clustering of Metagenomes (CCOM) tool that can be found at <http://edwards.sdsu.edu/ContigClustering/>. This web based tool requires an input of all the metagenomes per project along with the assembled contig file. The input reads file must not be the “*AllReads.fasta*” file, but the individual metagenomes that were concatenated to form the *AllReads* file. The reads must have a **.fna* extension, and the contig with the **.fasta* extension.

The resulting output can be downloaded to a local computer which contains all the intermediary outputs, .*bam*, and *.bai* files for all the reads), along with three folders with results from three binning tools; crAss, GroopM, and MetaBat. CrAss provides a table with the number of reads from the different metagenomic read files to form a contig. GroopM folder includes a list a of fasta files containing contigs belonging to one bin. Similarly, MetaBat folder contains a list of fasta files that contain contigs belonging to

1. Population genomes identification was performed using FOCUS. This tool can be downloaded from <https://sourceforge.net/projects/metagenomefocus/>. All the MetaBat bins were placed in a folder which was provided as input to FOCUS. The resulting output provides the taxa profile of each bin within the folder.

*python focus.py -q <Contigs and Reads containing folder > -s 1 -l all -o <output file>*

Bins containing most of its sequences belonging to particular taxa was identified to be a population genome. An additional step to assess the completeness of the bin can be performed using CheckM. CheckM requires additional programs to be installed to run CheckM, follow the steps from <https://github.com/Ecogenomics/CheckM/wiki/Installation>. To determine the genome quality, follow the commands from <https://github.com/Ecogenomics/CheckM/wiki/Genome-Quality-Commands>.

1. Phylogenetic affiliations of the reconstructed novel population genomes were predicted using three tools; CheckM, PhyloSift and RAST. First novel genomes were identified as bins that contained sequences belonging to specific taxa, but had high genome completeness (CheckM).
   1. CheckM to calculate the genome completeness, simultaneously looks for the 43 phylogenetically informative markers to place the bin in a bacterial genome tree. Presence and absence of 43 markers identifies the closest neighbor of the bins

The tree and tree-qa command from <https://github.com/Ecogenomics/CheckM/wiki/Genome-Quality-Commands> can be used to identify the sister lineages of the bin.

- 1. PhyloSift tools calculates the phylogenetic diversity of metagenomic samples. The tool looks for phylogenetically informative markers per sample to identify the sample and their closest neighbors. PhyloSift can be downloaded from <https://phylosift.wordpress.com/tutorials/running-phylosift/>. The pipeline and the commands are listed out in their manual <https://phylosift.wordpress.com/tutorials/running-phylosift/>. The option “*all”* in the command runs all the tests. The input sequence must be each novel bin.

*./phylosift all <bin.fasta>*

The resulting output is a directory with a tree (*.jplace*), taxasummary.txt, markersummary.txt (<https://phylosift.wordpress.com/tutorials/phylosift-outputs/>). “taxasummary.txt” file lists out the different taxa identified per bin with a score next them in a descending order. The best hit will therefore be the first taxa listed in this file.

- 1. RAST is a Rapid annotation of sequences using a subsystem technology, where the bins can be uploaded to the RAST server. The sequences are then annotated first using the tRNA scan, to identify all the RNA sequences per bin. Then based on presence of the RNA, closest neighbors are selected. Further, all the protein encoding genes within bin are compared against the selected closest neighbors giving them a score.

First step will be to request an account using the following link <http://rast.nmpdr.org/rast.cgi?page=Register>. The password will be mailed to your e-mail. Log in to your account and upload the novel population genomes to their servers. Finally, upon completion of the genome annotation pipeline, you can download the genes identified based on subsystems, compare genome against reference, and extract the list of closest neighbors.

Novel metagenome-assembled genome comparison against its phylogenetic neighbors identified by CheckM, PhyloSift, and RAST, using PATRIC. PATRIC is an online tool that can be found in <https://www.patricbrc.org/>. Create and account first using the <https://user.patricbrc.org/register>. Once you have an account, upload the novel population genome (bin) to PATRIC workspace. Then select “Services”, and go to “Annotation”. Upload the novel population genome as an input and annotate it against its phylogenetic neighbors sequentially. Then select “proteome comparison” under “Services” and select the annotated bins and phylogenetic neighbor as reference organisms to compare. The resulting output will be a proteome comparison graph, which can be downloaded along with
